# Bilateral muscle activation in postparalytic facial synkinesis: a cross-sectional high-resolution surface electromyography study

**DOI:** 10.1038/s41598-026-36015-1

**Published:** 2026-01-14

**Authors:** Paul F. Funk, Richard Schneider, Maren Schramm, Gerd Fabian Volk, Christoph Anders, Orlando Guntinas-Lichius

**Affiliations:** 1 Department of Otolaryngology, Jena University Hospital, Friedrich-Schiller- University Jena, Am Klinikum 1, 07747 Jena, Germany; 2 Division Motor Research, Pathophysiology and Biomechanics, Department of Trauma, Hand and Reconstructive Surgery, Jena University Hospital, Friedrich-Schiller-University Jena, Jena, Germany; 3 Facial-Nerve-Center Jena, Jena University Hospital, Jena, Germany; 4 Center for Rare Diseases, Jena University Hospital, Jena, Germany

**Keywords:** Neurophysiology, Musculoskeletal system, Muscle, Neurological disorders, Motor neuron disease

## Abstract

Facial synkinesis (FS) is a facial muscle coordination disorder following facial paralysis. We hypothesized that FS involves the entire network of facial muscles on both sides of the face. Bilateral facial high-resolution surface electromyography (HR-sEMG), using a total of 58 electrodes, was performed during 11 standardized facial movements in 36 healthy adult participants (53% female, 18–67 years) and 36 patients with FS (81% female, 24–70 years). Differences between the synkinetic and contralateral sides of patients and healthy controls were evaluated using linear mixed-effects models. Each movement task elicited a complex activation pattern involving nearly all measured facial muscles. In healthy controls, HR-sEMG activity was highest in the muscles primarily responsible for generating the intended voluntary movement. Notably, in patients with FS, muscles not normally expected to be strongly involved in a given movement showed markedly higher activity on the synkinetic side, and often also on the contralateral side, compared with healthy controls. Thus, facial movements in FS evoke a complex pattern of activity that only partially serves the intended movement and is further characterized by involuntary activation in muscles outside the primary movement region and on the contralateral side.

**Supplementary Information:**

The online version contains supplementary material available at 10.1038/s41598-026-36015-1.

## Introduction

Postparalytic facial nerve syndrome with synkinesis, hereafter referred to as facial synkinesis (FS), is a condition that follows severe facial nerve lesions with axonal damage leading to misguided nerve regeneration. FS is characterized by unintentional facial muscle activation and simultaneous mimic movements occurring alongside intentional facial actions^1^. Patients are often unable to fully control perioral and midfacial muscles, resulting in difficulties with eating, drinking, and facial expression^1^. Involuntary eye closure during intentional mouth movements, such as speaking or eating, is a disturbing symptom. This leads to both functional and aesthetic deficits, placing patients at risk for psychosocial sequelae and social impairment^2,3^.

To define optimal treatment, whether botulinum toxin therapy, physiotherapy, or surgery, a precise characterization of the synkinetically activated muscles is essential. However, no international standard for the clinical classification of FS currently exists. In practice, patients are often assessed by clinical examination, sometimes supported by grading systems such as the Sunnybrook or eFACE, which allow a regional description of synkinetic patterns but remain limited in scope^4,5^. The Synkinesis Assessment Questionnaire is an instrument developed specifically to capture this condition, but it is not widely used in routine clinical grading^6^. Electromyography (EMG) provides objective proof of synkinesis, usually via needle EMG, where activity is recorded in one muscle during voluntary contraction of another. While this approach can identify individual synkinetic patterns in detail, it is most often used to confirm oculo-oral synkinesis and is not routinely applied for broader characterization^7^.

HR-sEMG has been validated to capture multi-muscle facial activation with high classification accuracy and week-scale reliability in healthy adults^8–10^. Prior work in unilateral facial palsy indicates contralateral adaptations^11,12^. We therefore hypothesized that FS manifests as a bilateral network-level (i) dyscoordination on the synkinetic side and (ii) task-dependent, non-physiological activation on the contralateral side. Using two complementary HR-sEMG montages during 11 standardized expressions, we prespecified comparisons against healthy controls to examine both ‘expected’ (task-primary) and ‘unexpected’ (off-target) activations. Our aim was to provide a comprehensive overview of raw HR-sEMG activation patterns in FS as a basis for understanding network-level discoordination and for informing future research on individualized assessment and treatment approaches.

## Materials and methods

### Study design and setting

This was a cross-sectional observational study with a case-control comparison. The ethics committee of the Jena University Hospital approved the study (No. 2019 − 1539). All methods were performed in accordance with the relevant guidelines and regulations. The healthy control group was recruited at Jena University Hospital in September and October 2020. Patients were recruited at the same hospital between November 2021 and September 2022.

### Participants

The study included 36 patients (29 women) with a postparalytic facial nerve syndrome with synkinesis following prior peripheral facial palsy. Diagnosis was confirmed by facial electromyography^7^. Exclusion criteria included botulinum toxin treatment in the last 3 months, prior head or neck surgery specifically aimed at restoring facial function, other neurological or neuromuscular conditions, dermatologic conditions preventing electrode placement, and the presence of implanted electrical stimulators. Patients who received routine physiotherapy or speech therapy in the past were not excluded, but this variation in prior therapy is acknowledged as a limitation. All patients with a confirmed diagnosis who met the eligibility criteria were invited to participate prior to the initiation of a 10-day biofeedback therapy offered in the department. Those who agreed were recruited consecutively from the outpatient clinic of the Facial-Nerve-Center Jena, Department of Otorhinolaryngology, Jena University Hospital, Jena, Germany. Patient age ranged from 24 to 70 years. The median interval between onset of facial nerve palsy and the study was 24 months (range: 14 to 319 months). Etiologies included idiopathic facial palsy (*n* = 12), after tumor surgery (*n* = 11), Ramsay Hunt syndrome (*n* = 8), trauma (*n* = 2), and rare causes (*n* = 3). Clinical severity at baseline (Sunnybrook, eFACE, and patient-reported outcome measures) was assessed as part of a parallel study in the same patient cohort (Baum et al., 2025, submitted), but these measures were not used in the present analysis. Furthermore, the study included 36 healthy adult volunteers (19 women) with no neurological disease or history of facial surgery (age range: 18 to 67 years). Healthy controls were recruited from the general community (university staff, students, and local volunteers) using flyers and online announcements, representing a convenience sampling approach. The control cohort has been characterized in detail previously^8^. All participants gave written informed consent to participate in the study. Informed consent was also obtained for publication of information and images in an online open-access format.

### Variables

The primary quantitative variable was the RMS amplitude per electrode per task and side. The main categorical variables were group (patients vs. controls), side (synkinetic vs. contralateral), movement task, and electrode position.

The ‘synkinetic side’ was defined as the side previously affected by peripheral facial palsy, which consistently showed clinically evident synkinetic activation. The opposite side was therefore defined as the ‘contralateral’ side. This terminology does not imply that the contralateral side is unaffected, as discussed throughout the manuscript, but rather distinguishes it from the originally paralyzed side. Two patients had a history of facial palsy on the side defined as contralateral. In these cases, the contralateral side was excluded from analysis.

### Data sources and measurements

**Fig. 1:**
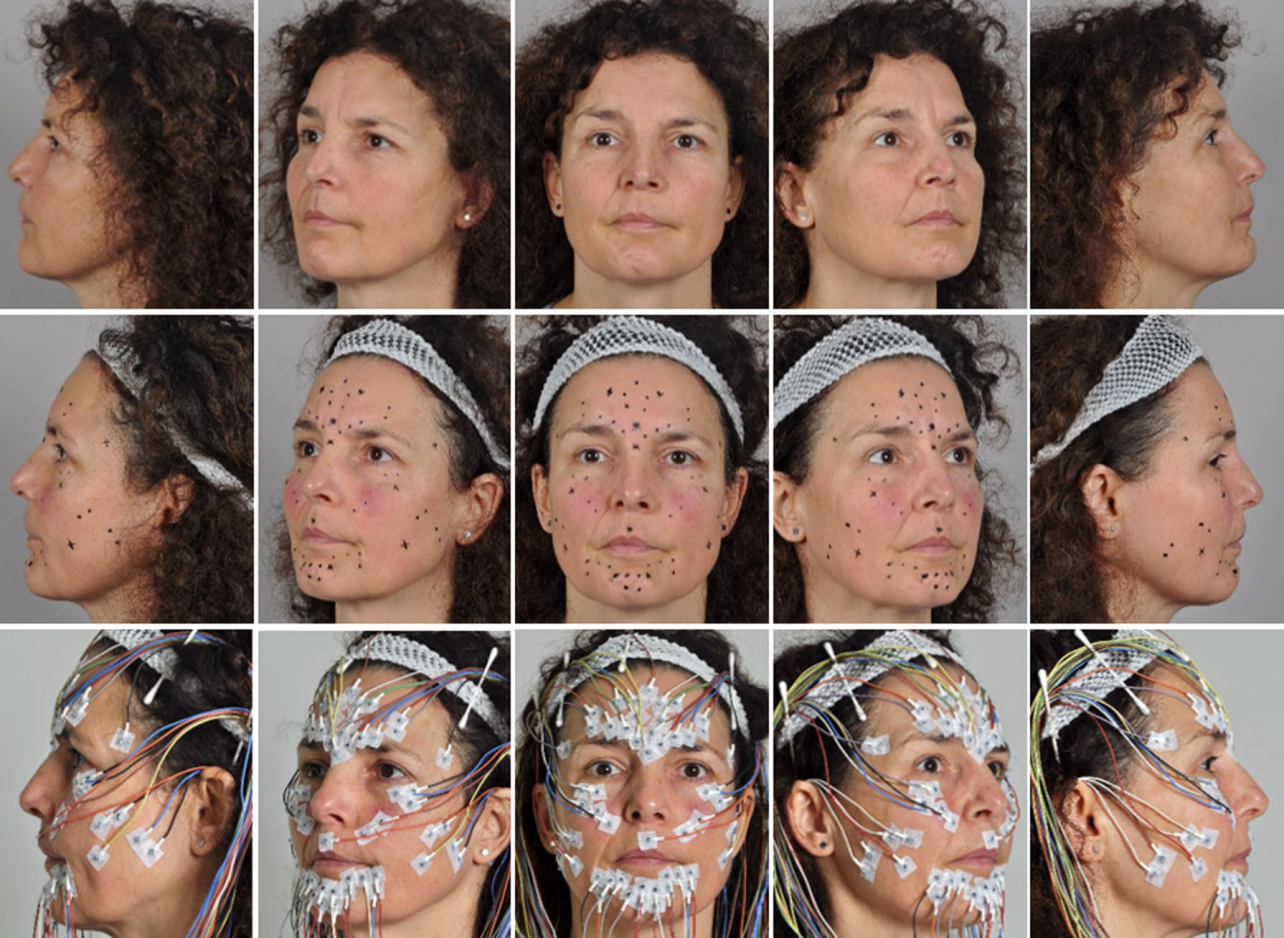
Standardized electrode marking and placement. Upper row: Example of a patient with synkinesis at rest. Middle row: Electrode positions for the Fridlund and Kuramoto schemes, marked using prefabricated stencils. Lower row: Complete surface EMG set-up with electrodes placed according to both schemes.

Surface electromyography was performed as described previously^8,9^, with high-resolution facial surface electromyography (HR-sEMG) recorded synchronously and symmetrically from both sides of the face using monopolar reusable Ag–AgCl disc electrodes (diameter 4 mm, DESS052606, GVB-geliMED, Bad Segeberg, Germany). Reference electrodes (disposable Ag–AgCl, H93 SG, Covidien, Neustadt, Germany) were placed bilaterally at the mastoid processes and connected. Two electrode arrangements were applied in parallel, following the schemes described by Fridlund and Cacioppo^13^ and by Kuramoto et al.^14^, with their positions illustrated in Fig. 1. The two electrode arrangements were labeled as the ‘Fridlund’ and ‘Kuramoto’ schemes. The Fridlund scheme follows the topography of the facial muscles, with eleven electrode pairs (inter-electrode distance: 1 cm) per hemiface, resulting in 21 electrodes per side, as two pairs shared one electrode. By contrast, the Kuramoto scheme does not consider muscle topography; instead, similar to an electroencephalogram (EEG), electrodes are positioned symmetrically at fixed inter-electrode distances^15^. This arrangement required 19 electrodes. Six electrodes were shared by both schemes, yielding a total of 58 electrodes placed on the face, including one ground and two linked reference electrodes.

HR-sEMG signals were recorded with a multichannel EMG system (gain: 100; frequency range: 10–1,861 Hz; sampling rate: 4,096/s; resolution: 5.96 nV/bit; DeMeTec, Langgöns, Germany). To minimize technical and physiological artifacts prior to analysis, the signals were centered and band-pass filtered between 10 and 500 Hz. In addition, a 50 Hz notch filter was applied to suppress interference from the electrical power supply^13^.

The exercises were performed as described previously^8,9^: The participants were instructed about the sequence of the examination and the facial exercises. Participants sat in a relaxed, upright position in front of a computer screen. They followed a self-explanatory video tutorial^16^, providing standardized, reliable instructions for facial movements, guided by a human instructor^17,18^. The instructor explained and demonstrated the following eleven facial expressions: Face at rest (R; no movement), wrinkling of the forehead (WF), closing the eyes normally (CEN), closing the eyes forcefully (CEF), wrinkling the nose (WN), closed mouth smiling (CMS), open mouth smiling (OMS), lip puckering (LP), blowing out the cheeks (BC), snarling (S), and depressing the lower lip (DLL). The participants performed each expression three times (three trials) before the next expression was explained. The sequence of movements was fixed and not counterbalanced. Each expression was done with an active contraction phase of approximately 5 s and a relaxation interval of about 2 s between repetitions. Short rest breaks were included after every few movements. All repetitions performed by the participants were retained for analysis. Performance was monitored in real time via a live camera feed by the examiner to ensure correct task execution. No formal artifact rejection or trial exclusion criteria were applied beyond visual verification of movement performance. The sequence of the facial movements is shown in Supplementary Figure S1.

### Bias

The standardized, video-guided instructions described earlier were used to reduce variability in performance. It is important to note that these instructions were not randomly ordered. The Fridlund scheme employs a monopolar montage during measurement; however, in the subsequent analysis, these data were processed in a bipolar manner^13^. This was achieved by subtracting the respective raw signals for each electrode pair and muscle. Such bipolar calculation provides spatial filtering and thus reduces crosstalk, but cannot completely exclude it.

This does not apply to the Kuramoto scheme, as the monopolar recordings were also analyzed as monopolar signals. Owing to the use of a monopolar montage, such data are inherently more prone to crosstalk. To reduce lateral interference, we connected the reference electrodes from both sides (positioned over the mastoid bones). This created a virtual reference electrode located at the center of the head. Since electrode differences in a monopolar montage are always calculated between the “active” and the reference electrode, only electrodes on the same side contribute as potential sources of crosstalk. As already noted, this cannot be completely excluded and represents a specific characteristic of monopolar montages. Nevertheless, the results obtained, which were largely consistent between the two montages, provide plausible indirect evidence that crosstalk did not substantially affect the data.

Potential confounders (sex, etiology, duration since onset) were not included in the mixed-effects model because the sample size did not allow reliable multivariable adjustment. However, these variables are acknowledged as relevant and will be considered in future larger-scale analyses.

### Study size

In preliminary investigations, strong effects were observed for both patients and healthy controls^8,9,19^. Therefore, the sample size calculation was based on an assumed effect size of 0.7. Under the assumption of a two-sided test, a global significance level of 0.05, and a power of 0.8, this resulted in a required sample size of 34 participants per group. Calculations were performed using G*Power software (version 3.1.9.4).

### Quantitative variables

For each of the three trials per movement task measured with HR-sEMG, a steady-state contraction phase was manually identified in the EMG signal. This segment was then divided into full 125-ms windows, and RMS amplitudes were computed for each window. Prior to statistical analysis, a plausibility check was performed to identify and remove outliers caused by technical artifacts. RMS amplitudes showed a right-skewed distribution, which is typical for EMG data. Therefore, outliers were defined using asymmetric, distribution-aware thresholds based on quartiles: Upper threshold = Q2 + 4 × (Q3 − Q2), Lower threshold = Q2 − 3 × (Q2 − Q1), where Q1 = lower quartile, Q2 = median, and Q3 = upper quartile. Values outside these thresholds were excluded before further processing. For each muscle/electrode and task, values were averaged across three trials (mean). No normalization to maximum voluntary contraction or resting baseline was applied, as the aim of the present analysis was to examine absolute activation patterns.

### Statistical methods

Linear mixed-effects model (LMM) statistics were applied as described previously^8,9^. For each scheme, a separate LMM was used to analyze the activation patterns of all electrodes. The LMM approach allows inclusion of both fixed and random effects, which elevates analyses for highly complex data^20^. HR-sEMG amplitudes for all facial expressions were first calculated as mean values with estimated 95% confidence intervals (CI). All electrodes were included in the LMM to evaluate the main effects of the parameters “group” (patients vs. controls), “movement task”, “side” (synkinetic vs. contralateral), and “electrode position”, together with their interactions. These were modeled as fixed effects. To account for repeated measures within subjects, a random intercept and subject ID were included as random effects, using an appropriate repeated covariance structure (CV) as implemented in SPSS (IBM SPSS Statistics 26). All predefined fixed effects and interactions were included in each respective model, no terms were removed based on statistical significance. We note that the pairwise comparisons were not performed in a fully exploratory fashion: all group contrasts were predefined at the level of individual muscles, sides, and movement tasks through structured filtering of the input data in Excel, ensuring hypothesis-driven comparisons. The pairwise comparisons were obtained using the least significant difference (LSD) procedure in SPSS, and the resulting p-values were adjusted for multiple comparisons using the Holm–Bonferroni method. The global significance level was set at 5%.

### Ethics approval

The ethics committee of the Jena University Hospital approved the study on October 19, 2019 (No. 2019-1539-BO). The study was not registered.

### Informed consent

Written informed consent was obtained from all participants to publish the information/image(s) in an online open access publication.

## Results

### Comparison of the facial muscle activation patterns between patients with facial synkinesis and healthy controls using the Fridlund HR-sEMG scheme

**Fig. 2:**
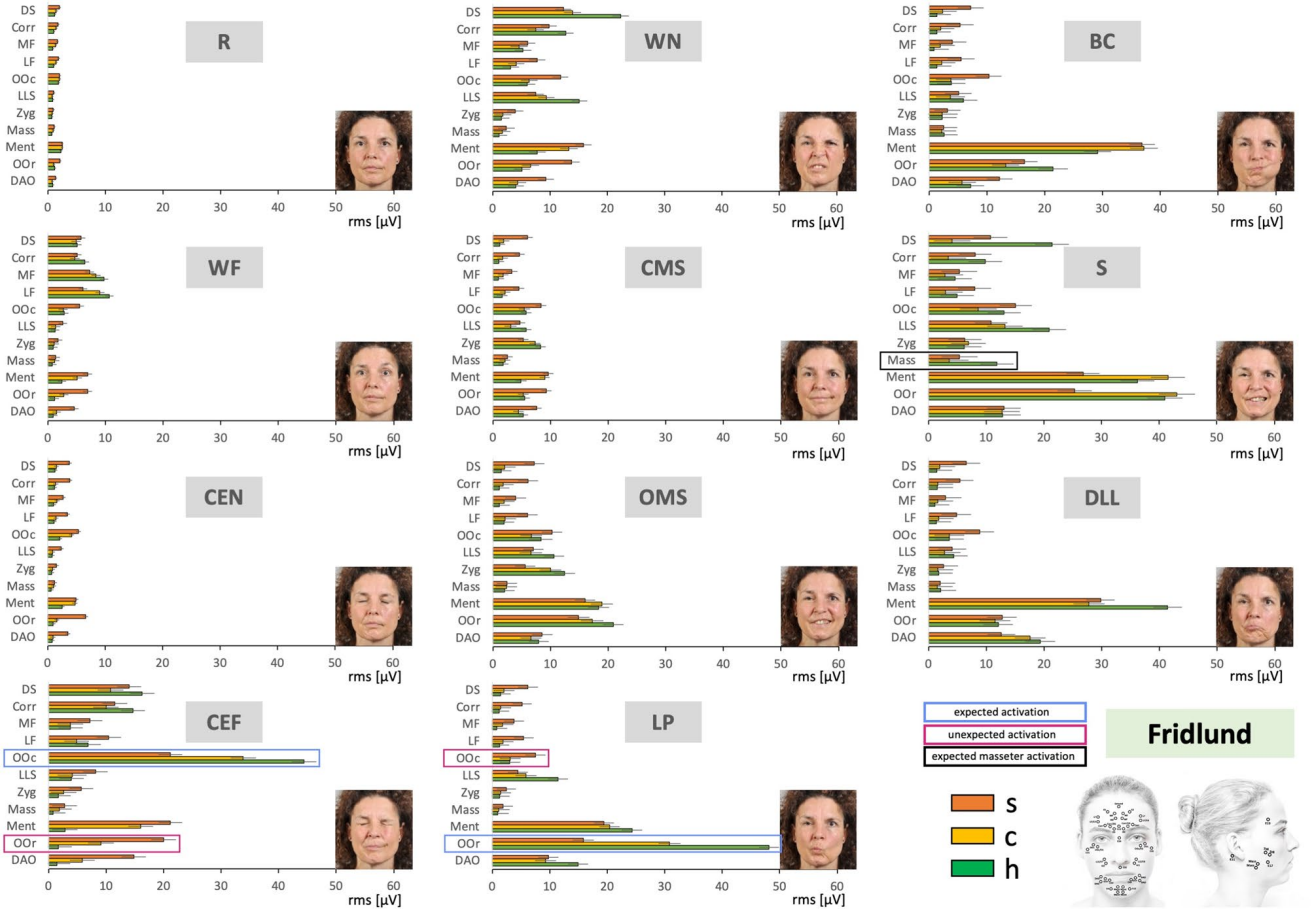
Average facial muscle activation recorded with the Fridlund HR-sEMG scheme during the sequence of 11 facial movement tasks from the upper left to the lower right. X-axis: facial muscle activation in root mean square (RMS, µV); mean ± 95% confidence interval. Y-axis: facial muscles from cranial to caudal. Orange = synkinetic side (upper bars); yellow = contralateral side (middle bars); green = healthy controls (lower bars). Blue boxes = areas expected to show high activity for the respective movement; magenta boxes = areas where high activity was not expected. Black box = expected masseter activation during snarling. Investigated facial muscles: *MF* frontal muscle, medial part; *LF* frontal muscle, lateral part; *Corr* corrugator supercilii muscle, *DS* depressor supercilii muscle, *OOc* orbicularis oculi muscle, *Zyg* zygomatic muscle, *LLS* levator labii superioris muscle, *Mass* masseter muscle (not innervated by facial nerve, control muscle), *OOr* orbicularis oris muscle, *DAO* depressor anguli oris muscle, *Ment* mentalis muscle. The facial movement tasks: *R* Face at rest, *WF* Wrinkling of the forehead, *CEN* Closing the eyes normally, *CEF* Closing the eyes forcefully, *WN* Wrinkling of the nose, *CMS* Closed mouth smiling, *OMS* Open mouth smiling, *LP* Lip puckering, *BC* Blowing out the cheeks, *S* Snarling, *DLL* Depressing lower lip.

Figure 2 summarizes the results of all recordings when using the Fridlund EMG scheme. Overall, the highest EMG activity was observed in the mentalis, orbicularis oris, orbicularis oculi, and depressor anguli oris muscles. In general, each movement task elicited activation in most or all measured facial muscles compared with rest. The masseter, which is innervated by the trigeminal nerve rather than the facial nerve, showed minimal activity during most movements but, as expected, exhibited strong activation during snarling, where clenching the teeth requires recruitment of chewing muscles (marked in Fig. 2 by a black box).

**Fig. 3:**
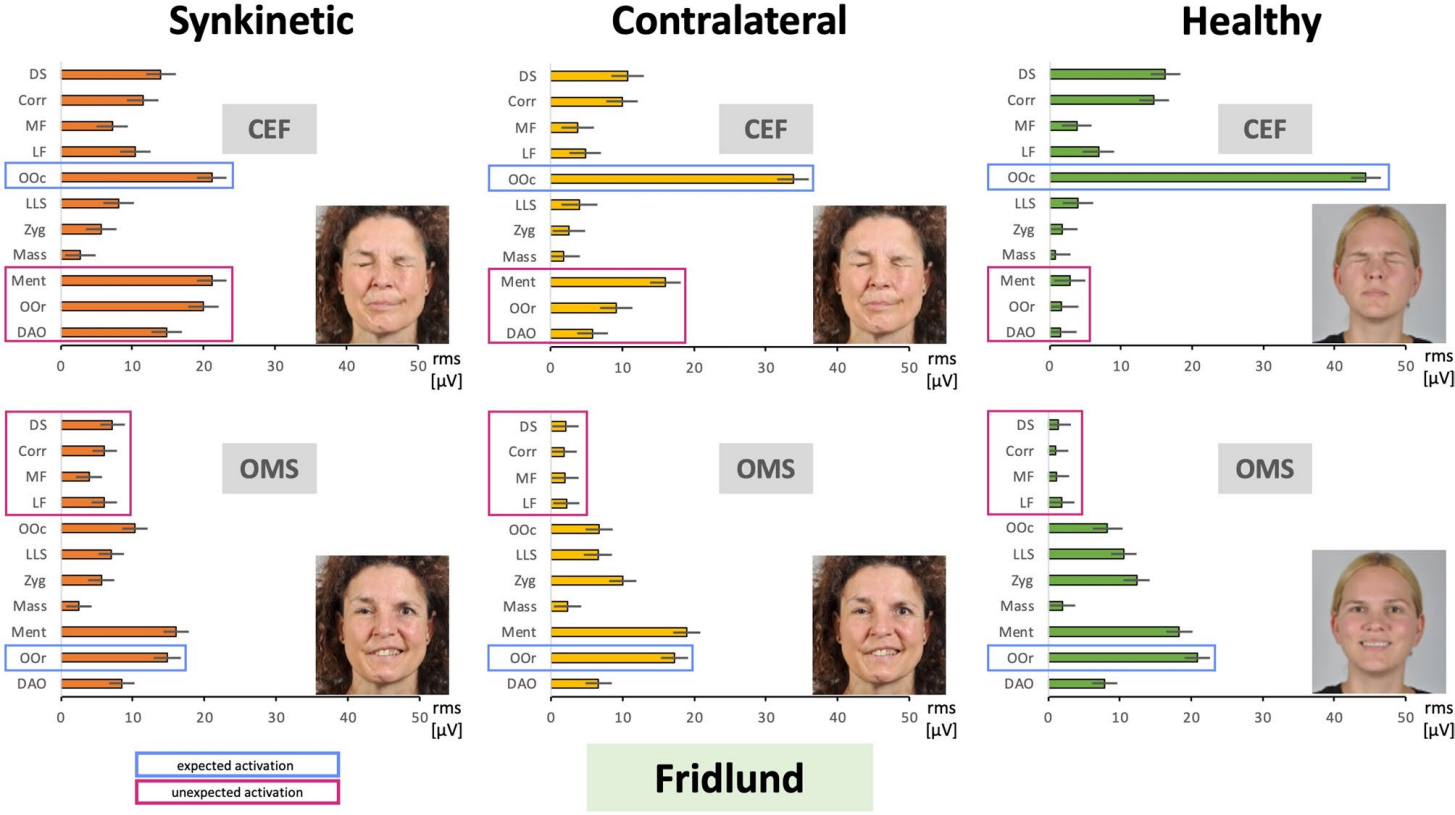
Two examples of facial movement tasks from Fig. 2 recorded with the Fridlund HR-sEMG scheme at higher resolution. Upper row: the task was to perform closing eyes forcefully (CEF). Lower row: the task was to perform open mouth smiling (OMS). X-axis: facial muscle activation in root mean square (RMS, µV); mean ± 95% confidence interval. Y-axis: facial muscles from cranial to caudal. The facial muscle activation is shown for the synkinetic side (left, orange), the contralateral side in patients (center, yellow), and for healthy controls (right, green). Blue boxes = areas expected to show high activity for the respective movement; magenta boxes = areas where high activity was not expected. Investigated facial muscles: *MF* frontal muscle, medial part; *LF* frontal muscle, lateral part; *Corr* corrugator supercilii muscle, *DS* depressor supercilii muscle, *OOc* orbicularis oculi muscle, *Zyg* zygomatic muscle, *LLS* levator labii superioris muscle, *Mass* masseter muscle (not innervated by facial nerve, control muscle), *OOr* orbicularis oris muscle, *DAO* depressor anguli oris muscle, *Ment* mentalis muscle.

The EMG activity was highest in facial muscles engaged in the voluntary movement, for example, in the eye region during eye closure or in the mouth region during lip puckering. Areas where high activity was expected for the respective movement are marked with a blue box in Fig. 2. Notably, activity in these regions was higher in healthy controls compared to patients with facial synkinesis. Conversely, on the synkinetic side, very high activity was also seen in areas where such strong activation would not be expected, for instance around the mouth during eye closure. Patients likewise often showed higher activity on the contralateral side in these regions compared to healthy controls. These areas, where high activity was not expected for the respective movement, are marked with a magenta box in Fig. 2. Figure 3 illustrates this in more detail for the task closing the eyes forcefully and for smiling with open mouth. As in Fig. 2, areas expected to show high activity are marked in blue, and areas where such activity was not expected are marked in magenta. In healthy controls, the orbicularis oculi muscle showed the highest activity during eye closure, with lower activation on both the synkinetic and contralateral sides of patients. On the synkinetic side, strong activity was also observed in the lower face, with similar increases visible on the contralateral side. A comparable pattern was evident for smiling with an open mouth: the orbicularis oris and zygomaticus muscles, which are expected to show high activity, were most active in healthy controls and less active in patients, with contralateral activity exceeding that on the synkinetic side. In contrast, upper-face muscles such as the frontalis, corrugator supercilii, and depressor supercilii, which are not expected to be strongly activated during this task, showed pronounced activation particularly on the synkinetic side. These visual patterns were supported by the statistical comparisons summarized in Table 1. Overall, facial muscle activation patterns differed significantly for most muscles across nearly all movement tasks between the synkinetic and contralateral sides, between the synkinetic side and healthy controls, and, for many tasks, also between the contralateral side and healthy controls. In addition, detailed results are provided in Supplementary Material S1 (SM1). Facial muscle activation is shown first as averages across all exercises, then for each exercise, and finally for each individual muscle. Statistical comparisons, corresponding to those summarized in Table 1, are displayed below each graph, with significant differences indicated by an asterisk. For example, in the task closing the eyes forcefully (SM1, Fig. 5 - CEF), healthy controls, the contralateral side, and the synkinetic side all differed significantly from each other in four muscles. During open mouth smiling (SM1, Fig. 8 – OMS), the synkinetic side was different from healthy controls in four of the five muscles marked in Fig. 3. However, the contralateral side showed an intermediate pattern: in some muscles (e.g., levator labii superioris) it did not differ significantly from the synkinetic side, in others (e.g., frontal muscle, lateral part; corrugator supercilii; depressor supercilii) it did not differ from healthy controls, and in one case (orbicularis oris) it was not significantly different from either group, lying between them in activation.

**Table 1:** Results of the statistical comparison of the HR-sEMG measurements with the Fridlund scheme for the different facial movement tasks and the activated facial muscles on the synkinetic side, the contralateral side, and in healthy controls.

Synkinetic side versus contralateral side in patients with synkinesis											
	MF	LF	Corr	DS	OOc	Zyg	LLS	Mass	OOr	DAO	Ment
R	*	*	*	**					***	***	
WF	*	***			***		**		***	***	***
CEN	***	***	***	***	***	*	***		***	***	
CEF	*	***		*	***	*	**		***	***	***
WN		***	*		***	*			***	***	*
CMS	*	***	***	***	***	***	*		***	***	
OMS		**	***	***	**	***					*
LP		**	**	***	***				***		
BC		*	*	**	***				*	***	
S		*	*	**	**				***		***
DLL			*	*	**					**	

Synkinetic side in patients versus healthy controls											
	MF	LF	Corr	DS	OOc	Zyg	LLS	Mass	OOr	DAO	Ment
R	***	***	***	***				**	***	**	
WF	***	***	*		***		*		***	***	***
CEN	***	***	***	***	***	**	***		***	***	***
CEF	*	*	*		***	*	**		***	***	***
WN		***	**	***	***	*	***		***	***	***
CMS	***	***	***	***	***	***			***	***	***
OMS	*	**	***	***		***	**		***		
LP	*	***	**	***	***		***		***	***	***
BC		*	*	***	***				**	**	***
S				***			***	**	***		***
DLL			*	**	**					***	***

Contralateral side in patients versus healthy controls											
	MF	LF	Corr	DS	OOc	Zyg	LLS	Mass	OOr	DAO	Ment
R	**	*	*								
WF	*	**	**						**		***
CEN					***				*		***
CEF			**	***	***				***	**	***
WN			***	***			***				***
CMS							***				***
OMS							**		**		
LP							***		***	***	**
BC									***		***
S			**	***	*		***	***			*
DLL											***

* *p* < 0.05; ** *p* < 0.01; *** *p* < 0.001; *R* Face at rest, *WF* Wrinkling of the forehead, *CEN* Closing the eyes normally, *CEF* Closing the eyes forcefully, *WN* Wrinkling of the nose, *CMS* Closed mouth smiling, *OMS* Open mouth smiling, *LP* Lip puckering, *BC* Blowing out the cheeks, *S* Snarling, *DLL* Depressing lower lip, *MF* frontal muscle, medial part; *LF* frontal muscle, lateral part; *Corr* corrugator supercilii muscle, *DS* depressor supercilii muscle, *OOc* orbicularis oculi muscle, *Zyg* zygomatic muscle, *LLS* levator labii superioris muscle, *Mass* masseter muscle (not innervated by facial nerve, control muscle), *OOr* orbicularis oris muscle, *DAO* depressor anguli oris muscle, *Ment* mentalis muscle.

**Fig. 4:**
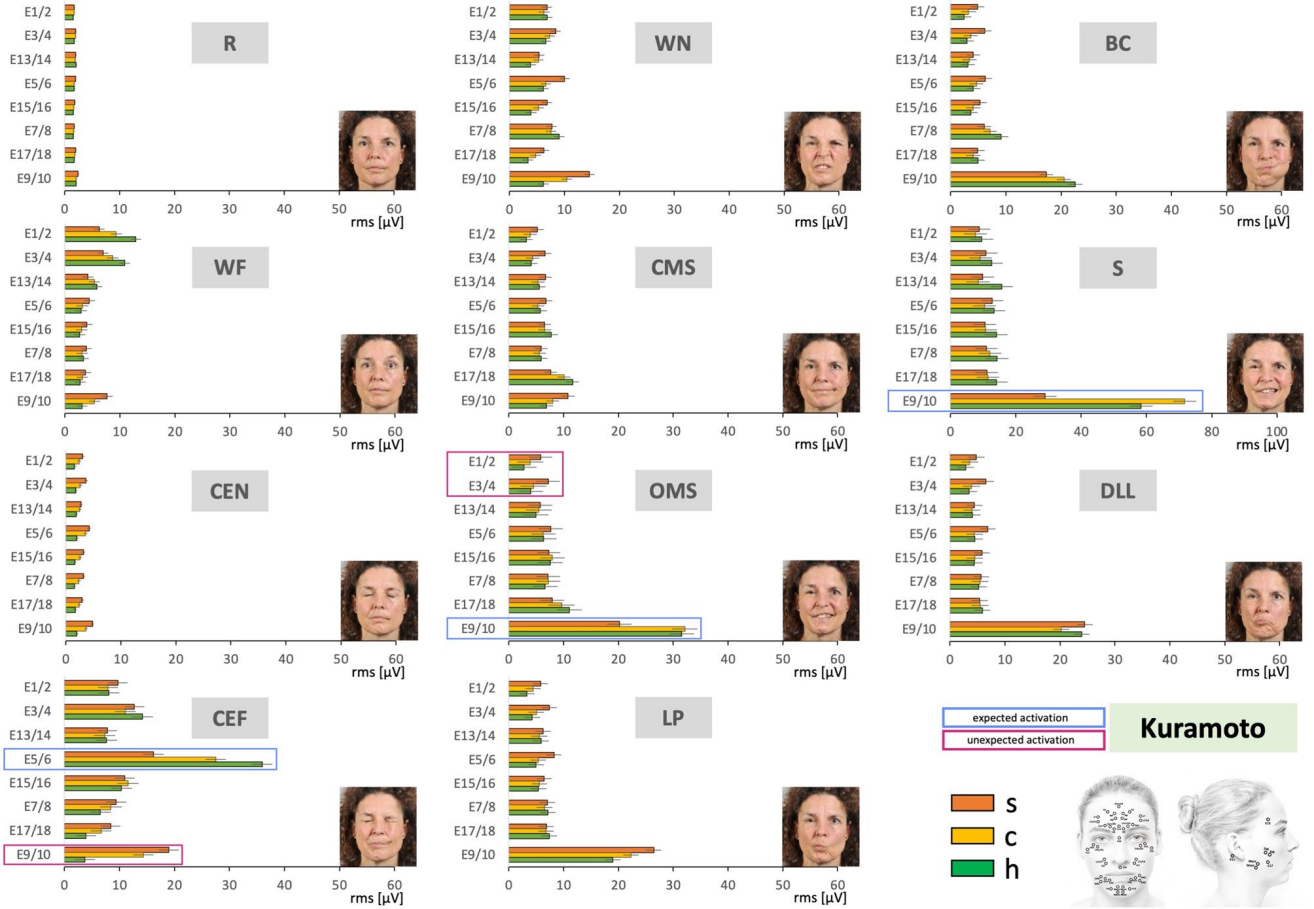
Average facial muscle activation recorded with the Kuramoto HR-sEMG scheme during the sequence of 11 facial movement tasks from the upper left to the lower right. X-axis: facial muscle activation in root mean square (RMS, µV); mean ± 95% confidence interval. Y-axis: electrode positions from cranial to caudal. Orange = synkinetic side (upper bars); yellow = contralateral side (middle bars); green = healthy controls (lower bars). Blue boxes = electrode positions expected to show high activity for the respective movement; magenta boxes = positions where high activity was not expected. Electrode positions: E1/2, E3/4, E5/6, E7/8, E9/10, E13/14, E15/16, E17/18 (Kuramoto scheme). The facial movement tasks: *R* Face at rest, *WF* Wrinkling of the forehead, *CEN* Closing the eyes normally, *CEF* Closing the eyes forcefully, *WN* Wrinkling of the nose, *CMS* Closed mouth smiling, *OMS* Open mouth smiling, *LP* Lip puckering, *BC* Blowing out the cheeks, *S* Snarling, *DLL* Depressing lower lip.

### Comparison of the facial muscle activation patterns between patients with facial synkinesis and healthy controls using the Kuramoto HR-sEMG scheme

**Fig. 5:**
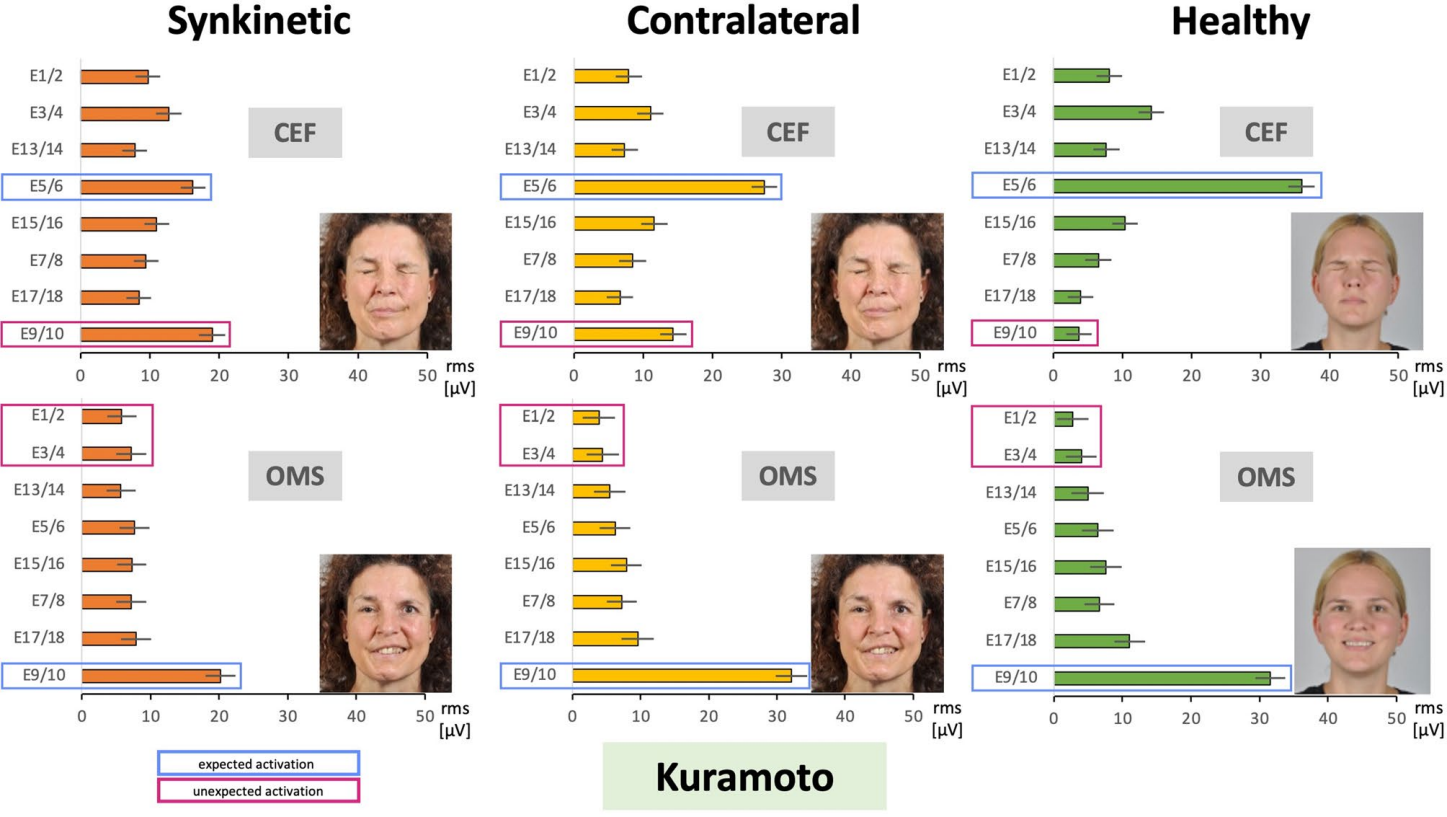
Two examples of facial movement tasks of Fig. 4 recorded with the Kuramoto HR-sEMG scheme at higher resolution. Upper row: the task was to perform closing eyes forcefully (CEF). Lower row: the task was to perform open mouth smiling (OMS). X-axis: facial muscle activation in root mean square (RMS, µV); mean ± 95% confidence interval. Y-axis: electrode positions from cranial to caudal. Facial muscle activation is shown for the synkinetic side (left, orange), the contralateral side in patients (center, yellow), and healthy controls (right, green). Blue boxes = positions expected to show high activity for the respective movement; magenta boxes = positions where high activity was not expected. Electrode positions: E1/2, E3/4, E5/6, E7/8, E9/10, E13/14, E15/16, E17/18 (Kuramoto scheme).

Figure 4 provides an overview of the recordings obtained with the Kuramoto EMG scheme. Overall, the results showed patterns similar to those observed with the Fridlund scheme. The highest EMG activity was again found in the mouth and eye regions. As with the Fridlund scheme, nearly all electrode positions were activated during each movement task. High sEMG activity was observed at electrode positions corresponding to muscle areas expected to show strong activation during the respective voluntary movement (some of these are again marked in blue). Just as in the Fridlund scheme, the activation in these expected areas was often higher in healthy controls than in patients. At the same time, consistent with the Fridlund results, elevated activity was observed on the synkinetic side in areas not expected to show high activation, with the contralateral side in patients likewise often exceeding activity in healthy controls (examples marked in magenta). Only in rare cases does the contralateral side deviate from the intermediate activation pattern also observed in the Fridlund scheme, e.g. during snarling, where it shows significantly higher activity than both the synkinetic side and healthy controls (highlighted by a blue box). Hence, the Kuramoto recordings revealed similar trends observed with the Fridlund scheme across movements. Figure 5 illustrates these findings for the same tasks shown with the Fridlund scheme (cf. Figure 3), namely closing the eyes forcefully (CEF) and smiling with an open mouth (OMS). In CEF, the highest activity was observed in the eye region, reflected by electrodes E5/6. Here, the activity was greatest in healthy controls, exceeding both the synkinetic and contralateral sides. Increased activation reflecting synkinetic activity was seen in the lower face, where electrodes E9/10 (marked in magenta boxes in Figs. 4 and 5) showed higher activity on the synkinetic side and, to a lesser extent, on the contralateral side compared with controls. Comparable patterns emerged in OMS. In an expected area, electrodes E9/10 exhibited the strongest activation in healthy controls and on the contralateral side, but reduced activity on the synkinetic side. Table 2 summarizes the statistical analysis for the Kuramoto recordings. Overall, activation patterns differed significantly for multiple electrode positions, between the synkinetic and contralateral sides, between the synkinetic side and healthy controls, and, in a few cases, also between the contralateral side and healthy controls. Statistically, the contrasts observed with the Kuramoto montage were generally less pronounced than those obtained with the muscle-specific Fridlund scheme. As for the Fridlund scheme, detailed results for the Kuramoto montage are provided in Supplementary Material S2 (SM2). The data are presented first as averages across all exercises, then for each exercise, and finally for each electrode position, with statistical comparisons shown below each graph and significant differences indicated by an asterisk. By examining, for example, the tasks highlighted above (SM2, Fig. 5 – CEF, Fig. 8 - OMS), it becomes evident that the most prominent activation differences described are statistically significant. Numerous additional examples in the supplementary material illustrate similar patterns, combining graphical and statistical information to allow a more detailed exploration of the findings.

**Table 2:** Results of the statistical comparison of the HR-sEMG measurements with the Kuramoto scheme for the different facial movement tasks and electrode positions on the synkinetic side, the contralateral side, and in healthy controls.

Synkinetic side versus contralateral side in patients with synkinesis								
	E1/2	E3/4	E13/14	E5/6	E15/16	E7/8	E17/18	E9/10
R				**	*		*	***
WF	***	**	*	*				***
CEN	***	***		***	***	***	***	***
CEF				***				***
WN		*		***	**		**	***
CMS	*	***	**	**			***	***
OMS								***
LP	*	***		*				***
BC		**		***				***
S								***
DLL		**		*				***

Synkinetic side in patients versus healthy controls								
	E1/2	E3/4	E13/14	E5/6	E15/16	E7/8	E17/18	E9/10
R		*					*	**
WF	***	***	*	*				***
CEN	***	***	***	***	***	***	***	***
CEF				***		*	***	***
WN		**	*	***	***	*	***	***
CMS	*	**					***	***
OMS		*					*	***
LP	**	***		*		***		***
BC	*	**		**				***
S			*					***
DLL		**		*				

Contralateral side in patients versus healthy controls								
	E1/2	E3/4	E13/14	E5/6	E15/16	E7/8	E17/18	E9/10
R								
WF	***	**						**
CEN	***	***	**	***	***	***	**	***
CEF		*		***			*	***
WN			*			*	*	***
CMS								
OMS								
LP						*		*
BC								**
S			**					***
DLL								***

* *p* < 0.05; ** *p* < 0.01; *** *p* < 0.001; *R* Face at rest, *WF* Wrinkling of the forehead, *CEN* Closing the eyes normally, *CEF* Closing the eyes forcefully, *WN* Wrinkling of the nose, *CMS* Closed mouth smiling, *OMS* Open mouth smiling, *LP* Lip puckering, *BC* Blowing out the cheeks, *S* Snarling, *DLL* Depressing lower lip.

## Discussion

The present study is, to our knowledge, one of the first to provide a detailed description of the complex patterns of disturbed facial muscle coordination in patients FS. Compared with previous EMG and MoCap studies that typically focused on selected facial regions or single tasks, the present study provides a bilateral characterization of facial muscle coordination across 11 standardized movements using two complementary HR-sEMG montages and linear mixed-effects modeling. This design enables the simultaneous analysis of synkinetic and contralateral activity patterns at high spatial resolution, thereby extending the scope of prior work that relied on single-muscle or unilateral assessments. While our cross-sectional design does not allow causal inference, the observed activation patterns provide important correlational evidence of the complex network involvement in FS. In practice, the intention to perform a specific facial movement was associated with synkinetic activity in essentially all facial muscles not directly involved in the voluntary task, with the exception of muscles not innervated by the facial nerve. Notably, the findings of this study were derived from a cohort of patients with widely varying disease durations. Synkinesis after facial nerve palsy remains a persistent burden for many patients and can impair quality of life in both motor and psychosocial domains, even decades after the initial paralysis^21,22^. To date, no long-term studies have demonstrated that synkinesis spontaneously becomes substantially milder over time, although some patients may perceive their symptoms as less disturbing when adapting their behavior by avoiding certain movements^22^. This observation is consistent with the underlying pathomechanisms: the development and persistence of synkinesis are facilitated by misdirected nerve regeneration (collateral sprouting) and incomplete remyelination, leading to individual axons innervating multiple muscle groups and thereby producing involuntary co-activations. Ephaptic transmission and increased excitability of the facial motor nucleus may further contribute^7^. These central and peripheral alterations usually persist for many years. However, surgical and rehabilitative interventions can attenuate the development and severity of synkinesis, and successful therapy is often associated with a long-term reduction in symptom burden^23,24^.

Equally important is the recognition that the contralateral side also shows abnormal muscle activation, including in muscles not closely related to the intended movement. The consistent patterns observed with the Fridlund scheme indicate that the contralateral side does not simply resemble either healthy controls or the synkinetic side. Instead, depending on the task and muscle group, contralateral activity was sometimes closer to healthy controls and sometimes to the synkinetic side, while in some cases it lay in between. This suggests that the contralateral side may operate in an “intermediate state,” reflecting both its original activation pattern and adaptive adjustments toward the synkinetic side. Although our cross-sectional data cannot confirm this, these findings may be consistent with a central coordination strategy that synchronizes both facial nerves to maintain overall facial symmetry, even if this comes at the cost of introducing maladaptive activity.

In general, multiple physiological mechanisms have been reported that may also account for contralateral involvement. One possibility is contralateral reinnervation of denervated muscle fibers, particularly in midline-adjacent muscles such as the frontalis or orbicularis oris, through aberrant or cross-nerve sprouting^25^. This process results in the contralateral nerve innervating more muscle fibers than before, thereby altering the innervation pattern. In addition, following acute facial palsy, the excitability of motoneurons and interneurons in the brainstem circuits controlling facial musculature increases. This leads to elevated baseline tone and sometimes excessive activity on the non-paralyzed side^22^. Functional MRI studies have shown cortical reorganization following facial nerve dysfunction^12^. Three-dimensional facial movement analyses have further demonstrated that the mobility of the contralateral side is restricted in patients with unilateral palsy, indicating that it is functionally constrained and cannot reach its full range of motion^26^. Hyperactivity of contralateral muscles may develop to compensate for these limitations, thereby accentuating asymmetry and exaggerating facial expressions^27^. With longer disease duration, such hyperactivity can become chronic, potentially leading to structural and functional changes in the muscles^27^. Over time, some patients restrict their facial expressiveness to minimize unwanted contractions, which may further influence overall facial activity and social communication^22^. These mechanisms underline why the non-paralyzed side should be referred to as the ‘contralateral’ rather than a ‘healthy’ side.

The Kuramoto montage also showed less significant differences especially for lower face movements compared with Fridlund. This may be explained by methodological differences. The Fridlund montage uses bipolar derivations, which act as a spatial filter and provide greater specificity for localizing muscle activity. In contrast, the Kuramoto montage is based on monopolar recordings, which are more susceptible to crosstalk from adjacent muscles and integrate signals over larger regions. Its coverage of the lower face is also sparse, limited to E9/10 near the mouth and E17/18 at the margin of the masseter. Other positions, such as the philtrum electrode (E20), were excluded for comparability, as midline electrodes cannot be assigned to a specific side and are not included in the Fridlund layout. By contrast, the Fridlund montage includes twelve electrode positions per side, capturing activity from multiple lower-face muscles. This denser spatial sampling likely explains why lower-face differences appeared more clearly in the Fridlund results.

Although our analysis focused on differences between patients and healthy controls, it is important to note that physiological asymmetries in facial muscle activation may also occur in healthy individuals. In our prior work on healthy controls, no significant side differences were detected^8^, consistent with previous studies reporting largely symmetrical activation^14,28–30^. We therefore averaged both healthy hemifaces to establish a single normative reference. However, task dependency appears to play a role: while most voluntary facial movements are symmetrical, some degree of lateralization has been observed in high-density EMG during phonation tasks^31^. This natural variability needs to be taken into account when interpreting pathological patterns. While the present study descriptively distinguishes between “expected” and “unexpected” activation patterns based on typical muscle involvement in specific facial expressions, we did not formalize these into composite indices. This decision reflects the current lack of standardized muscle activation maps for facial expressions and the resulting ambiguity in assigning clear regional boundaries. Future studies with larger cohorts and refined anatomical and physiological priors may help define quantitative indices of symmetry and off-target activation more systematically.

The present findings underscore the value of bilateral HR-sEMG as a method capable of providing detailed insights into facial muscle activation. Earlier studies have demonstrated that HR-sEMG can characterize the complex recruitment patterns of the entire facial muscle network during both functional movements and emotional expressions^8,9,30,32^. A single-muscle activation during functional tasks is the exception rather than the rule. Dual-channel needle EMG may be sufficient to demonstrate the presence of facial synkinesis^7,33^, but it does not allow for a comprehensive characterization of the disorder. In clinical assessments, synkinesis is typically described in a bidirectional manner, with the intended muscle group named first, and followed by the unintended muscle group. For example, oculo-oral synkinesis refers to oral commissure movement occurring during voluntary eye closure. Conversely, oral-oculo synkinesis describes involuntary eye closure during voluntary oral movement^34–36^. Such classifications are based on direct visual inspection or video analysis. The Synkinesis Assessment Questionnaire is structured in the same way^6,37^. It records involuntary movement in one facial region during voluntary movement in another. The synkinesis subscore of the Sunnybrook facial nerve grading system, in turn, evaluates only the severity (visibility) of synkinesis during specific facial tasks^4,38^. Automated image analysis approaches likewise focus only on specific regions, for example, by quantifying relative movements between facial areas or by automatically measuring eye surface changes during movement tasks^39–41^. The limitation of all these methods lies in their reliance on purely visual analysis. On this basis, it has even been suggested that certain types of synkinesis are more common than others, or that some patients show, for example, no synkinesis in the upper face during lip puckering^35,41^. Our findings challenge conclusions drawn from purely visual assessments and highlight that synkinesis may be more widespread than previously assumed. HR-sEMG provides a more detailed and less invasive approach for characterizing facial muscle activity than approaches such as electrical stimulation for facial nerve mapping, which we proposed in earlier work^42,43^.

Nevertheless, the present study only focuses on the internal dimension of facial muscle activation as captured by HR-sEMG, and it is important to recognize that other assessment tools, such as video- and motion-capture (MoCap)–based systems, provide an external perspective by quantifying visible facial displacements^44–46^. HR-sEMG captures the timing and specificity of individual muscle activations with high temporal resolution, but it does not directly measure the resulting mechanical motion^10^. In contrast, MoCap systems quantify the spatial displacement of facial landmarks and overall symmetry, yet they are limited by occlusion, marker interference, and their inability to infer the underlying muscle source of movement. Integrating both modalities would therefore allow the dissociation of electrical activation from mechanical output, offering a more complete characterization of synkinetic activity. To address the challenge of electrode-related occlusion in video recordings, we recently introduced electromyography-informed facial expression reconstruction (EIFER)^47^. This approach leverages a 3D morphable model combined with neural image-to-image translation to restore occluded facial regions and link HR-sEMG activity with facial geometry. It provides a foundation for future multimodal analyses that integrate internal muscle activity with external kinematics to achieve a more comprehensive profile of facial motor dysfunction.

Accurate identification of both intended and unintended muscle activations is essential for providing meaningful symptomatic relief in patients with facial synkinesis^36,48^. Current treatment options to improve facial expression include chemodenervation with botulinum toxin, physical or EMG-based biofeedback therapy, and surgical interventions such as neurectomy or myectomy^49,50^. These therapies are all directed at areas of unintended facial muscle activity. Chemodenervation is sometimes applied on the contralateral side to improve facial asymmetry, and weakening of the depressor labii inferioris muscle has also recently been proposed^23,51^. Treatment outcomes are generally more favorable when interventions are tailored to the individual patient^52^. To date, treatment outcomes for synkinesis have primarily been evaluated using the aforementioned tools and patient-reported outcome measures^53,54^. We propose that with further refinement, HR-sEMG could be used not only to characterize the individual synkinesis pattern of patients with facial palsy, but also to assess therapy, as recently demonstrated by Schneider et al.^19^. The present study further demonstrates that HR-sEMG could also enable tailoring of treatment on the contralateral side, as it reveals involvement of many more muscles than previously recognized.

### Limitations

In this study, patients were measured at one time point. Therefore, the reliability and variability of the observed activation patterns in patients with respect to classical test–retest validation remain unknown. Following participation in the present study, however, the same patients underwent a 10-day biofeedback training program for synkinesis, the results of which we recently published^19^. These data demonstrated that repeated HR-sEMG measurements were sufficiently precise to capture therapy-induced changes in activation patterns on both the synkinetic and contralateral sides. Nevertheless, a dedicated test–retest validation study in a patient cohort without therapeutic intervention is still warranted, and we aim to address this in future research, as we have previously done in the healthy control group^9^.

The facial tasks were performed in a fixed order to ensure standardized instruction and comparability across participants. However, this design does not rule out potential order or fatigue effects, which may have influenced activation levels across tasks.

This study focused on general amplitude differences between patients with facial synkinesis and healthy controls. No normalization was applied to account for individual or movement-dependent amplitude variation. Previous analyses of this cohort included maximum-normalized comparisons to further characterize individual movement profiles^8,19^.

A noteworthy aspect is the gender imbalance in our patient cohort (81% female). Prior studies in healthy controls suggest that sex differences in facial sEMG activity exist, with women often showing stronger and more emotionally reactive responses, particularly in muscles such as the zygomaticus major and corrugator supercilii^55–57^. However, these findings are muscle- and task-dependent and less consistent for voluntary movements^56^. For patients with facial synkinesis, no systematic analyses of sex-specific activation patterns have been reported to date. Our cohort size and imbalance did not permit reliable modeling of sex as a covariate. Similarly, the etiology and duration of post-paralytic facial syndrome varied widely across patients and the distribution was too heterogeneous to allow a meaningful analysis of those factors. Treatment status also differed widely and was not formally controlled. Most patients had received physiotherapy or speech therapy as part of routine clinical care in the past. Therefore, residual effects of prior treatments cannot be fully excluded. While these factors may influence individual activation patterns, our primary aim was to characterize general group-level differences between patients and healthy controls. Future work with larger datasets will be needed to disentangle such contributions and define more personalized activation models.

As mentioned in the Methods section, surface electromyography is prone to some degree of crosstalk from adjacent muscles, particularly in monopolar montages. Although the results of the present study, and a recent analysis published by our group, indicate that the observed patterns reflect true physiological activity of distinct facial regions, the precise extent to which crosstalk contributes to the HR-sEMG signals cannot be fully determined^19^. Nevertheless, it remains essential to examine the raw signals in this context, as they illustrate what HR-sEMG measurements directly capture and provide the necessary foundation for interpreting more complex analytical approaches. Future work should therefore incorporate advanced signal processing techniques, such as independent component analysis (ICA), to further disentangle the complexity of the facial muscle network. Recent work by Man et al. provides a first demonstration of the information that can be extracted by such an ICA-based approach in healthy adults^58^.

In this study, reference electrodes were linked across both mastoids, creating a virtual central reference. This configuration minimizes lateral bias and is common in bilateral EMG setups. However, alternative strategies such as common average (CAR) or Laplacian referencing may yield slightly different spatial potential distributions and could be explored in future work to test robustness of observed activation patterns.

Finally, the two EMG schemes applied in this study were originally developed for psychophysical and emotion research and therefore did not include the platysma. As the platysma can be markedly affected in facial synkinesis^59^, extending HR-sEMG coverage to this muscle would be a worthwhile direction for future studies.

## Conclusions

Facial synkinesis involves the entire network of facial muscles and should not be regarded as a disorder limited to a few specific muscles. Rather, it represents a generalized discoordination of facial muscle activity. Importantly, synkinesis on one side of the face also alters activation patterns on the contralateral side, which differ from those seen in healthy controls. These findings emphasize that optimal treatment strategies should consider not only the synkinetic side but ideally also address involvement of the contralateral side.

## Supplementary Information

Below is the link to the electronic supplementary material.

Supplementary Material 1

Supplementary Material 2

Supplementary Material 3

## Data Availability

The original contributions presented in the study are included in the article/Supplementary material, further inquiries can be directed to the corresponding author/s.
