# Supplementary material for: Bilateral muscle activation in postparalytic facial synkinesis: a cross-sectional high-resolution surface electromyography study
Source: Sci Rep. 2026 Jan 14;16:2057. doi: 10.1038/s41598-026-36015-1 (PMC12808638; doi:10.1038/s41598-026-36015-1)
Supplement: Supplementary file 1 — Supplementary Material 1 [file 41598_2026_36015_MOESM1_ESM.pdf]

## Supplementary Material for the Fridlund Scheme

On the following pages, results are presented first as averages across all exercises, then for each exercise, and finally for each facial muscle. Facial muscle activation is shown as root mean square (RMS) values in µV with mean ±95% confidence intervals. Statistical comparisons (corrected for multiple testing) are provided below each graph, with significant differences indicated by an asterisk (* $p<0.05$; ** $p<0.01$; *** $p<0.001$, Holm–Bonferroni corrected) .

Abbreviations:

**Investigated facial muscles**: MF = frontal muscle, medial part; LF = frontal muscle, lateral part; Corr = corrugator supercilii muscle; DS = depressor supercilii muscle; OOc = orbicularis oculi muscle; Zyg = zygomatic muscle; LLS = levator labii superioris muscle; Mass = masseter muscle (not innervated by facial nerve, control muscle); OOr = orbicularis oris muscle; DAO = depressor anguli oris muscle; Ment = mentalis muscle.

**The facial movement tasks**: R = Face at rest; WF = Wrinkling of the forehead; CEN = Closing the eyes normally; CEF = Closing the eyes forcefully; WN = Wrinkling of the nose; CMS = Closed mouth smiling; OMS = Open mouth smiling; LP = Lip puckering; BC = Blowing out the cheeks; S = Snarling; DLL = Depressing lower lip.

**Facial sides**: s = synkinetic side (orange); c = contralateral side (yellow); h = healthy controls (green)

# Electrode schemes

## Fridlund electrodes abbreviated with related muscle names

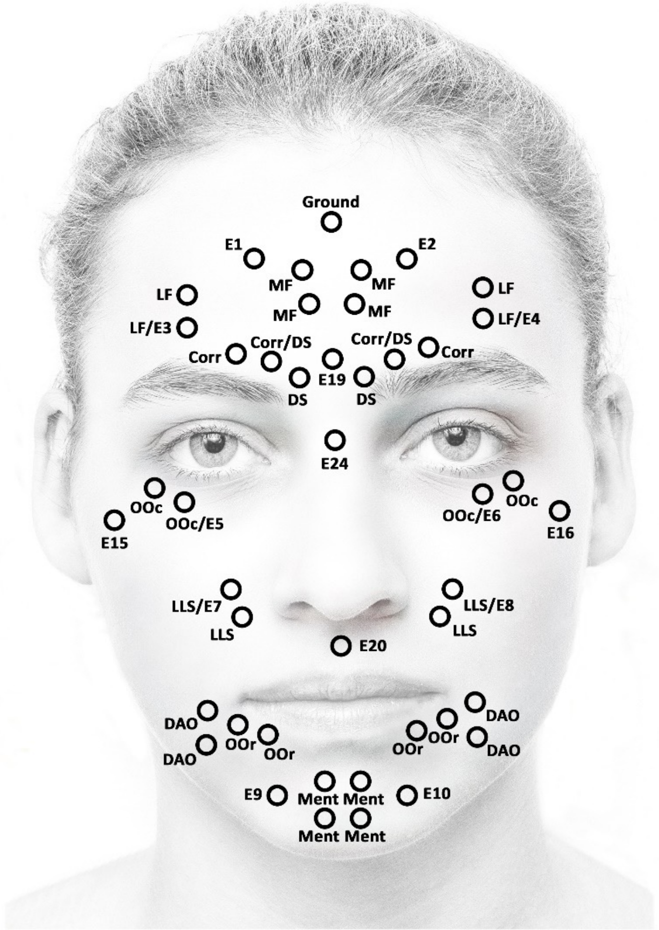


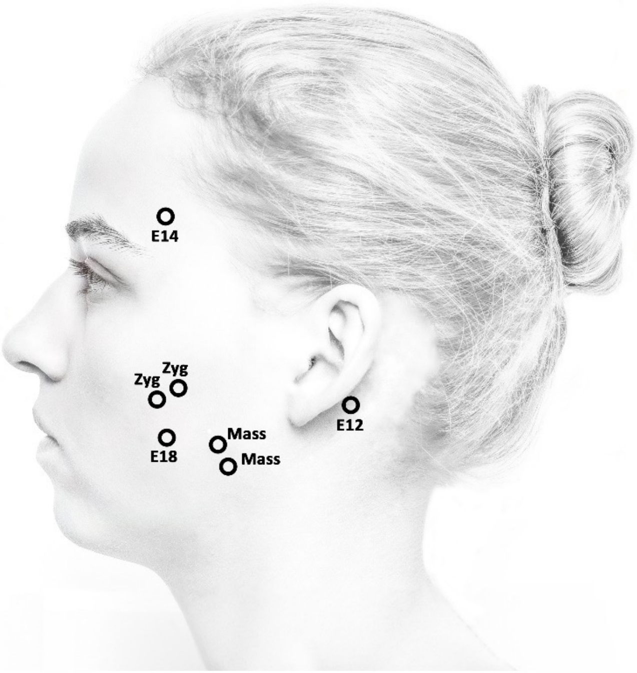


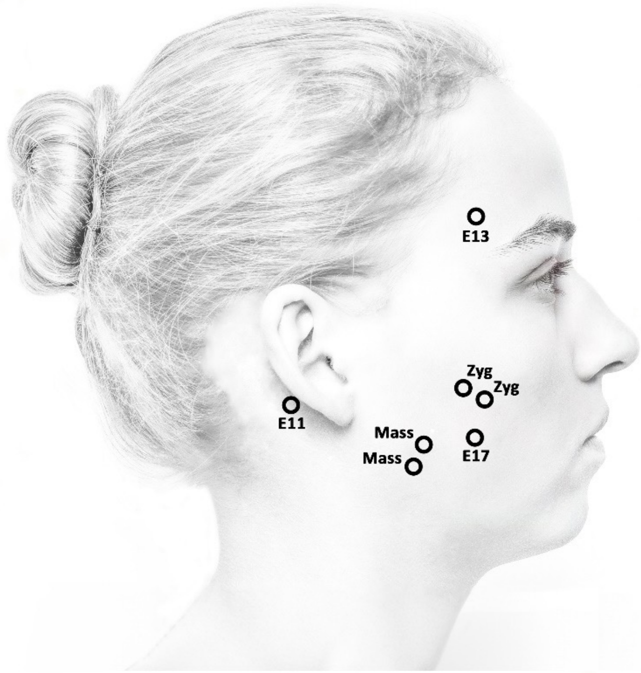

# 1 - Independent of facial expression

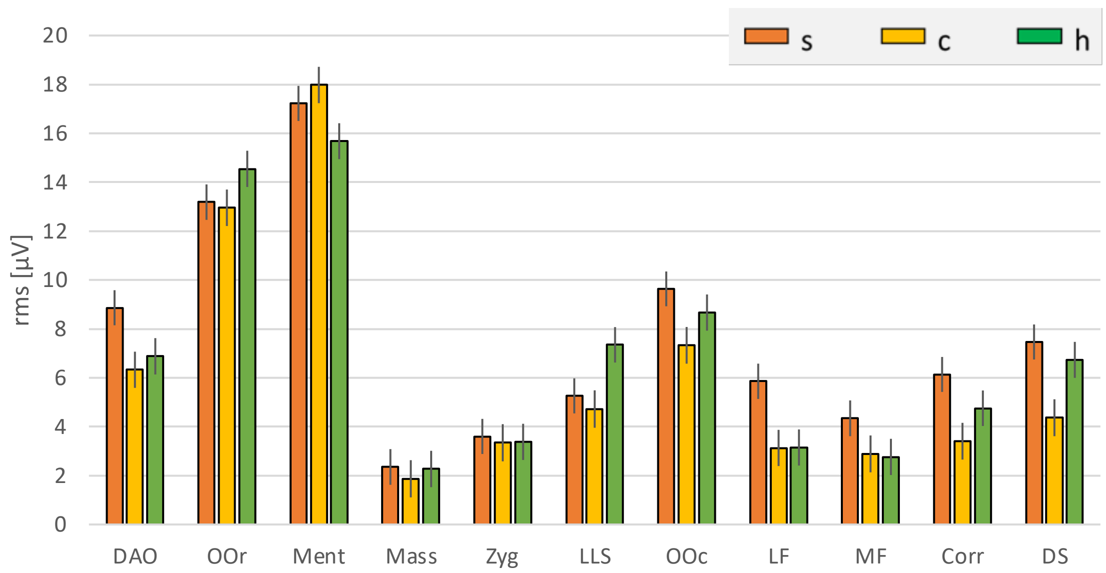


| | | | | | | | | | | | |
|---|---|---|---|---|---|---|---|---|---|---|---|
| s vs. c | *** | | | | | | *** | *** | ** | *** | *** |
| s vs. h | ** | | * | | | *** | | *** | * | * | |
| c vs. h | | * | *** | | | *** | | | | | *** |

# 2 - R

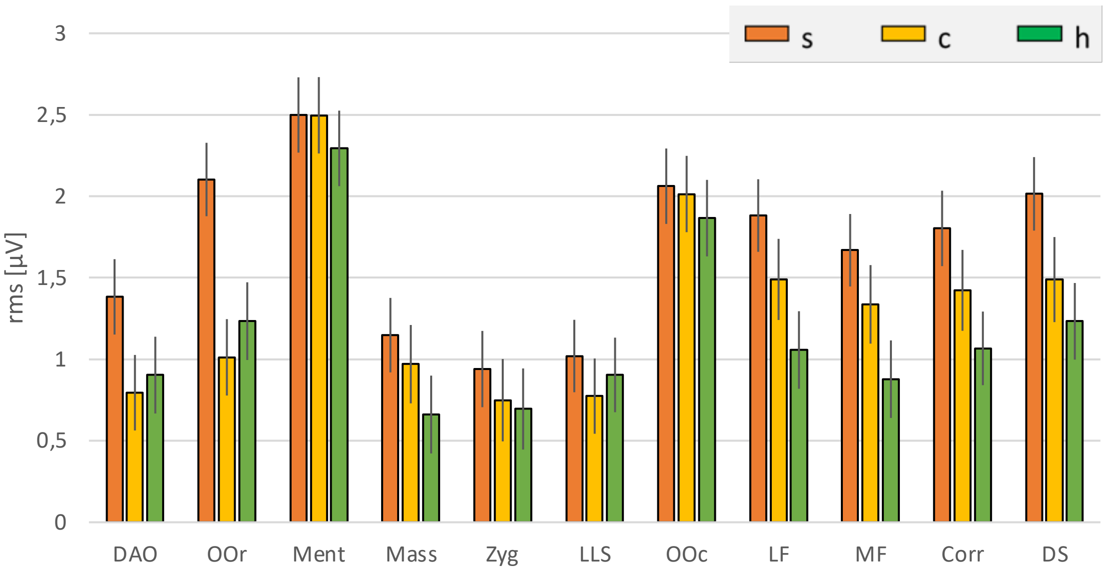


| | | | | | | | | | | | |
|---|---|---|---|---|---|---|---|---|---|---|---|
| s vs. c | ** | *** | | | | | | | | | * |
| s vs. h | * | *** | | * | | | | *** | *** | *** | *** |
| c vs. h | | | | | | | | | | | |

# 3 - WF

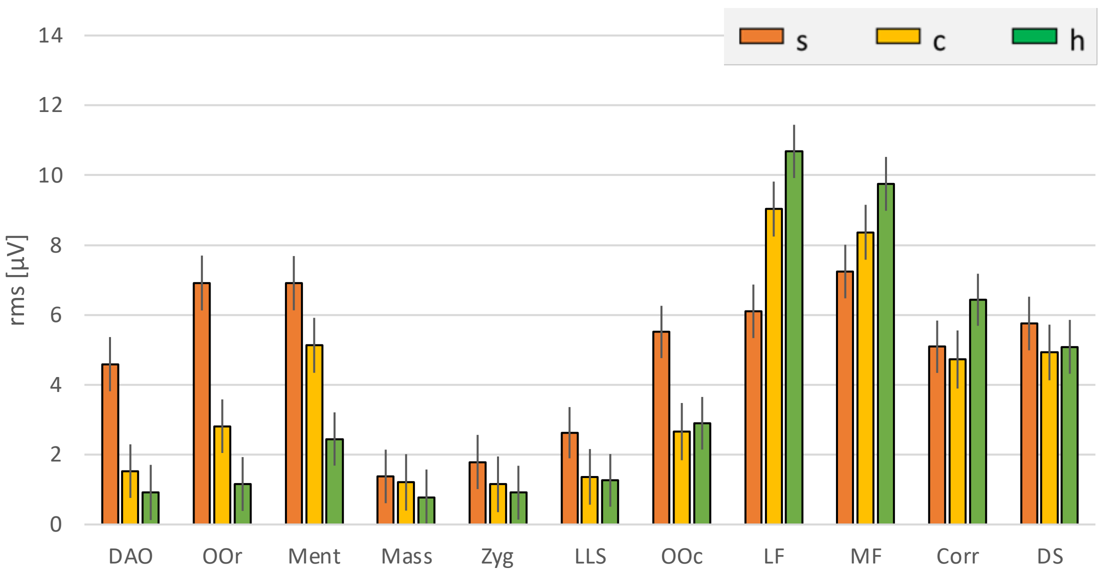


| | | | | | | | | | | | |
|---|---|---|---|---|---|---|---|---|---|---|---|
| s vs. c | *** | *** | ** | | | | *** | *** | | | |
| s vs. h | *** | *** | *** | | | | *** | *** | *** | | |
| c vs. h | | * | *** | | | | | * | | * | |

# 4 - CEN

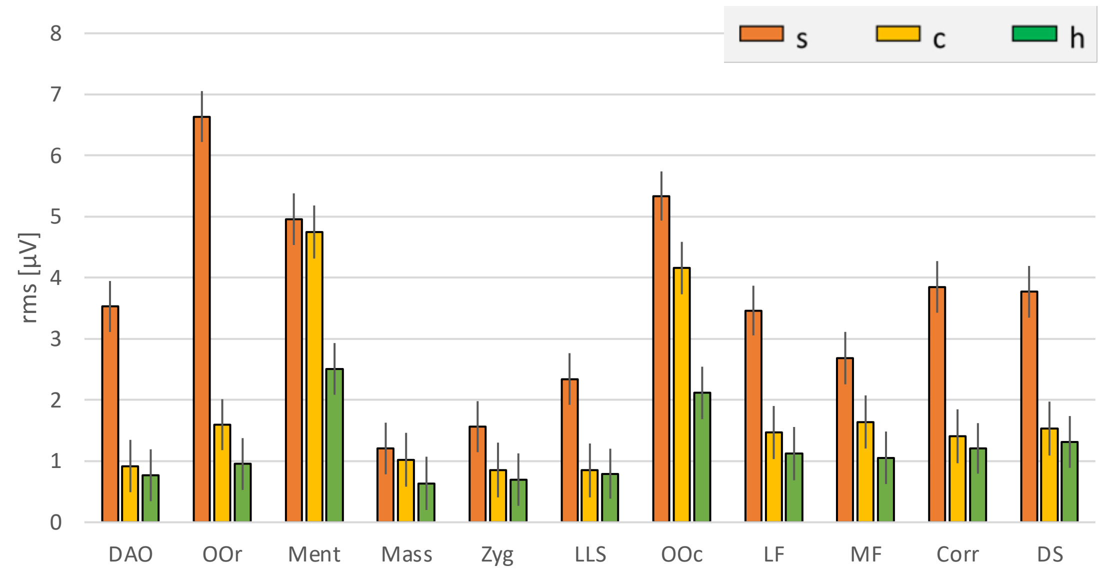


| | | | | | | | | | | | |
|---|---|---|---|---|---|---|---|---|---|---|---|
| s vs. c | *** | *** | | | * | *** | *** | *** | ** | *** | *** |
| s vs. h | *** | *** | *** | | ** | *** | *** | *** | *** | *** | *** |
| c vs. h | | | *** | | | | *** | | | | |

## 5 - CEF

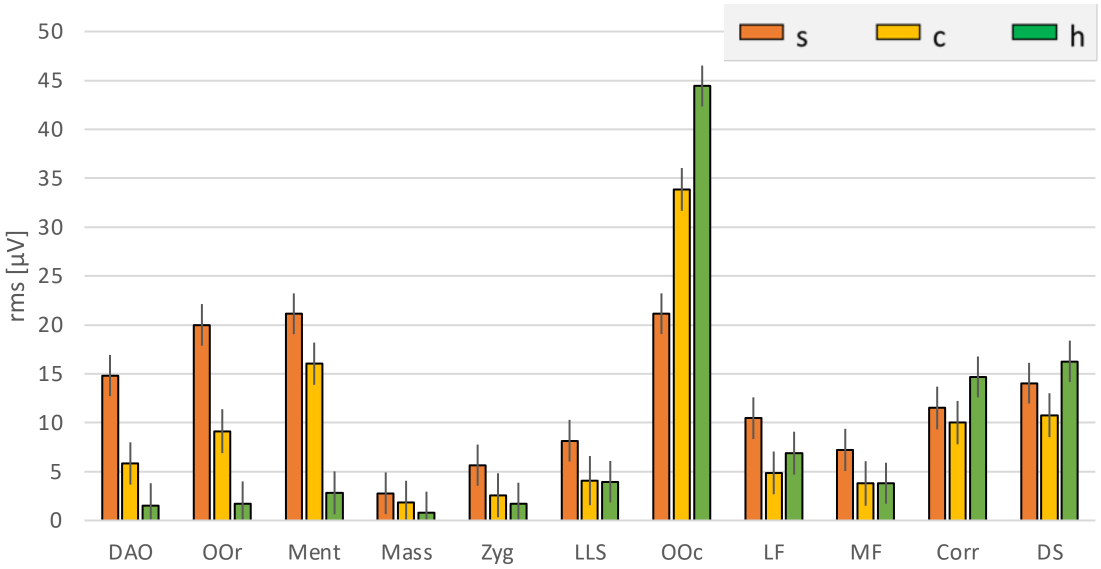


| | | | | | | | | | | | |
|---|---|---|---|---|---|---|---|---|---|---|---|
| s vs. c | *** | *** | ** | | | * | *** | *** | | | |
| s vs. h | *** | *** | *** | | | * | *** | | | | |
| c vs. h | * | *** | *** | | | | *** | | | * | ** |

## 6 - WN

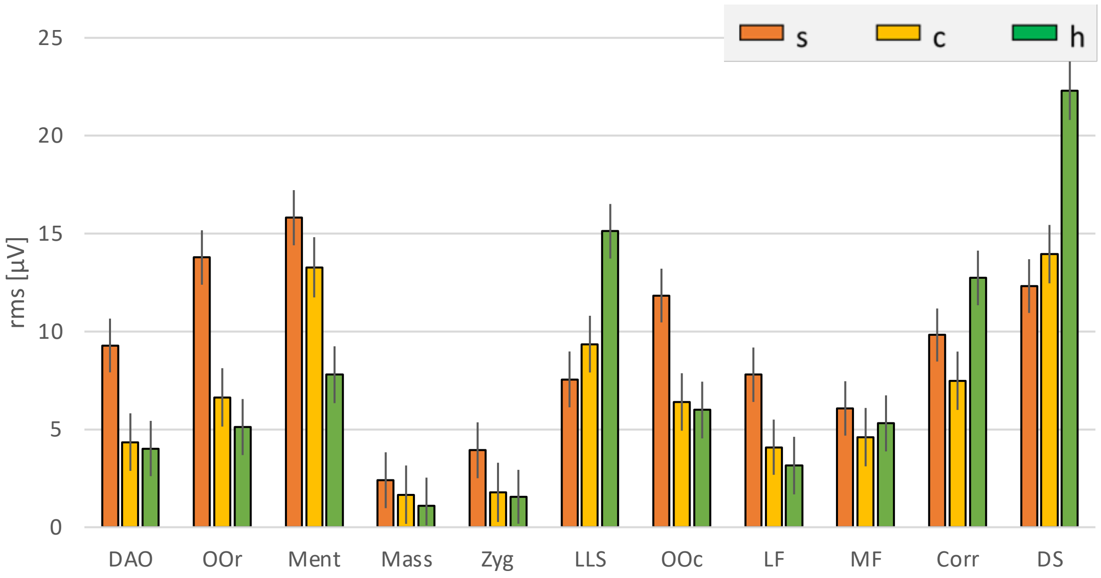


| | | | | | | | | | | | |
|---|---|---|---|---|---|---|---|---|---|---|---|
| s vs. c | *** | *** | | | | | *** | *** | | | |
| s vs. h | *** | *** | *** | | | *** | *** | *** | | * | *** |
| c vs. h | | | *** | | | *** | | | | *** | *** |

## 7 - CMS

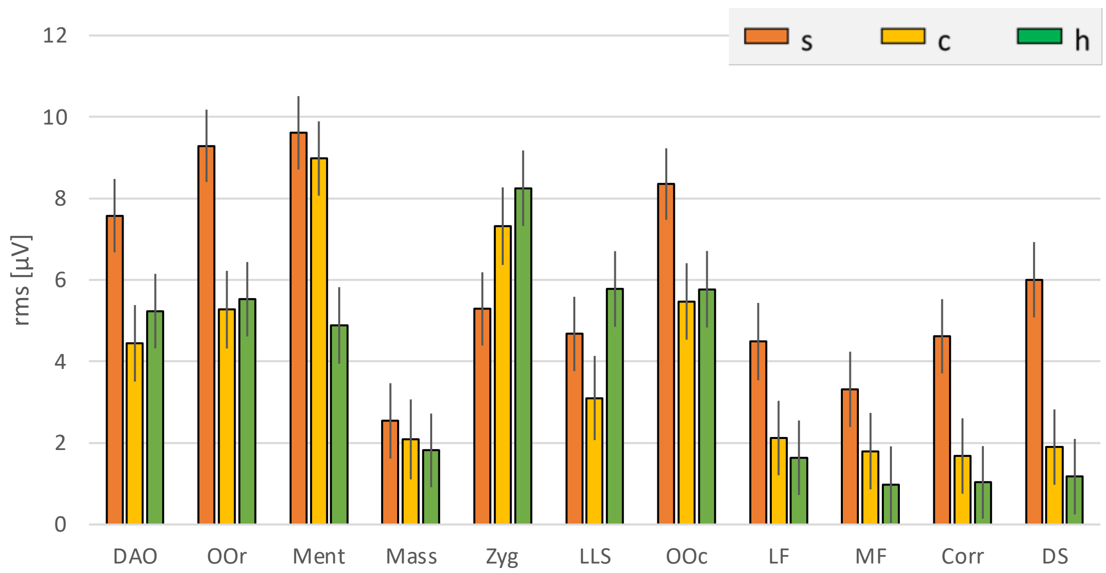


| | | | | | | | | | | | |
|---|---|---|---|---|---|---|---|---|---|---|---|
| s vs. c | *** | *** | | | ** | * | *** | *** | | *** | *** |
| s vs. h | ** | *** | *** | | *** | | *** | *** | ** | *** | *** |
| c vs. h | | | *** | | | ** | | | | | |

## 8 - OMS

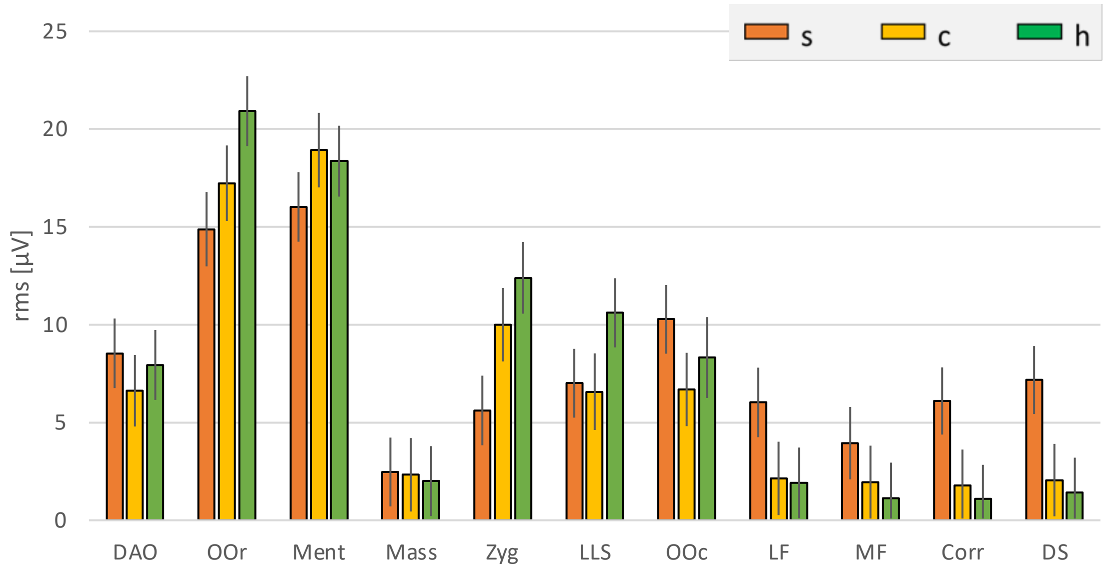


| | | | | | | | | | | | |
|---|---|---|---|---|---|---|---|---|---|---|---|
| s vs. c | | | | | ** | | * | ** | | ** | *** |
| s vs. h | | *** | | | *** | * | | * | | *** | *** |
| c vs. h | | | | | | * | | | | | |

# 9 - LP

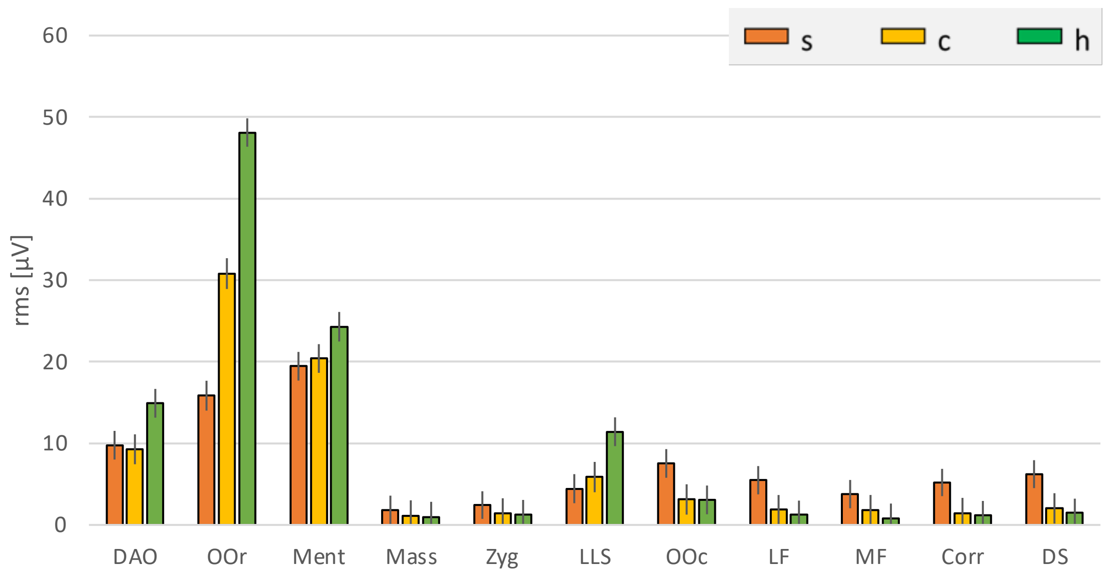


| | | | | | | | | | | | |
|---|---|---|---|---|---|---|---|---|---|---|---|
| s vs. c | | *** | | | | | ** | * | | * | ** |
| s vs. h | *** | *** | ** | | | *** | ** | ** | | ** | ** |
| c vs. h | *** | *** | * | | | *** | | | | | |

# 10 - BC

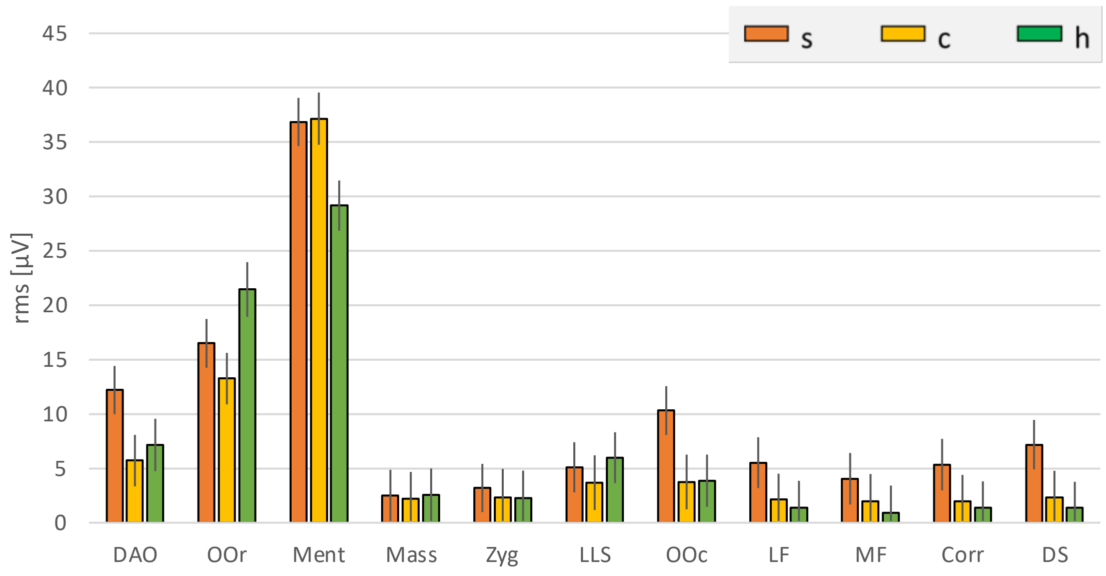


| | | | | | | | | | | | |
|---|---|---|---|---|---|---|---|---|---|---|---|
| s vs. c | *** | | | | | | *** | | | | * |
| s vs. h | * | * | *** | | | | ** | | | | ** |
| c vs. h | | *** | *** | | | | | | | | |

# 11 - S

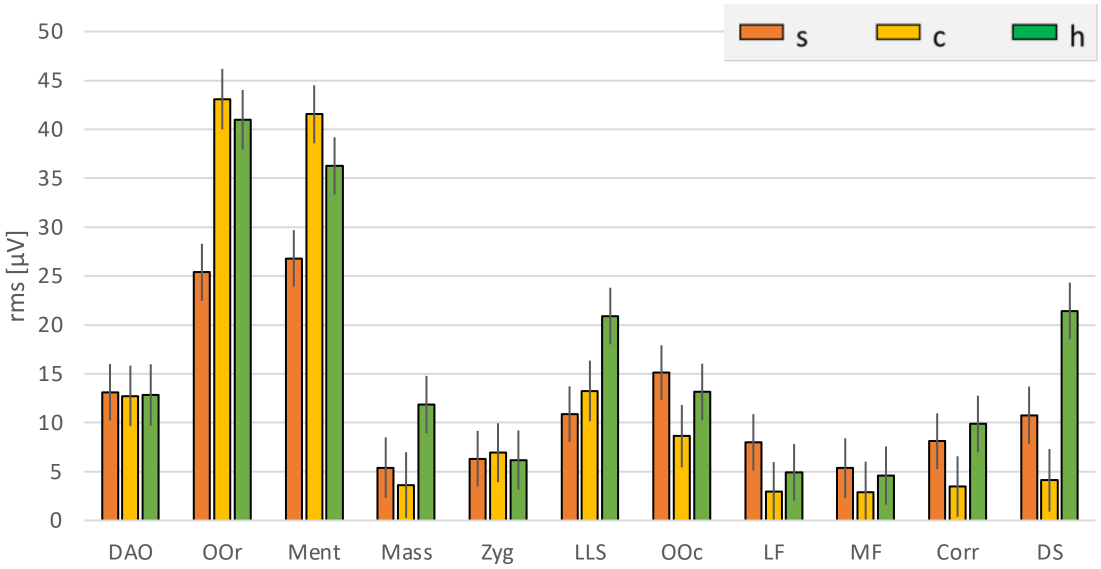


| | | | | | | | | | | | |
|---|---|---|---|---|---|---|---|---|---|---|---|
| s vs. c | | *** | *** | | | | * | | | | ** |
| s vs. h | | *** | *** | * | | *** | | | | | *** |
| c vs. h | | | | ** | | ** | | | | * | *** |

# 12 - DLL

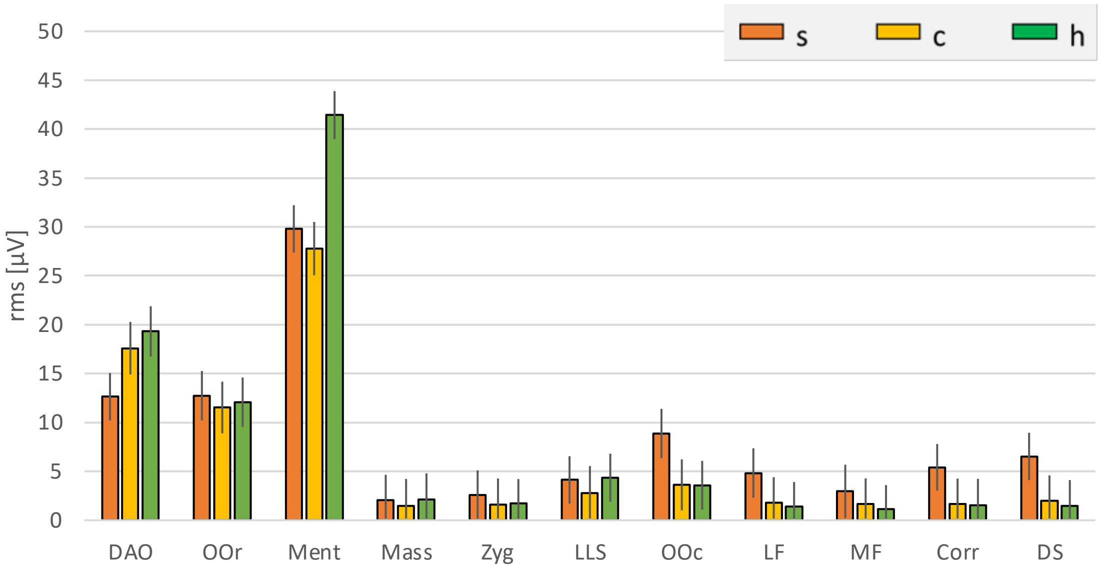


| | | | | | | | | | | | |
|---|---|---|---|---|---|---|---|---|---|---|---|
| s vs. c | | | | | | | * | | | | |
| s vs. h | ** | | *** | | | | * | | | | * |
| c vs. h | | | *** | | | | | | | | |

# 13 - Independent of electrode position

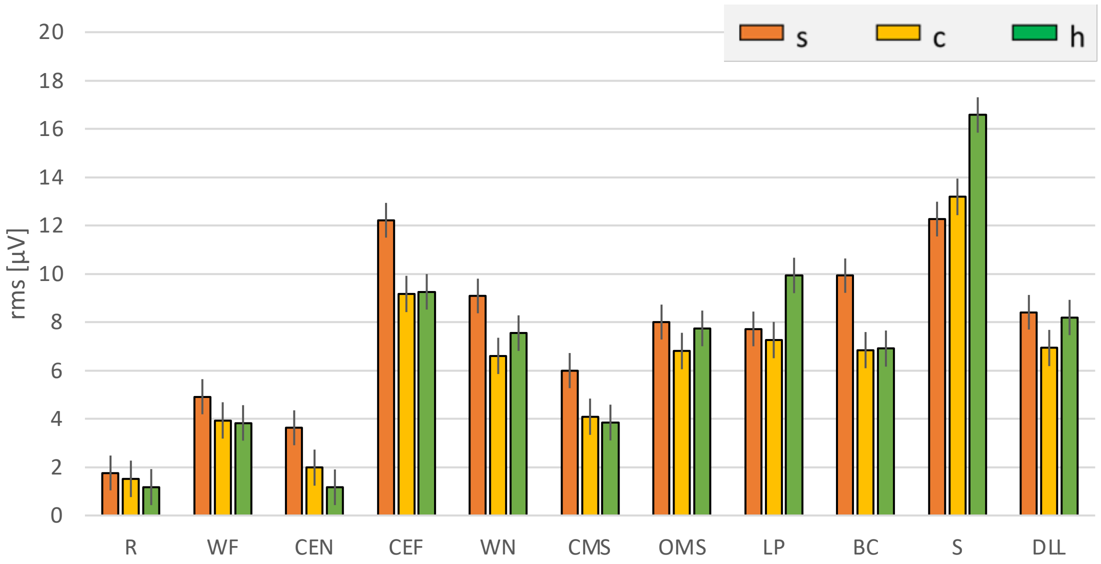


| | | | | | | | | | | | |
|---|---|---|---|---|---|---|---|---|---|---|---|
| s vs. c | | | *** | *** | *** | *** | * | | *** | | ** |
| s vs. h | | | *** | *** | * | *** | | *** | *** | *** | |
| c vs. h | | | | | | | | *** | | *** | |

# 14 - DAO

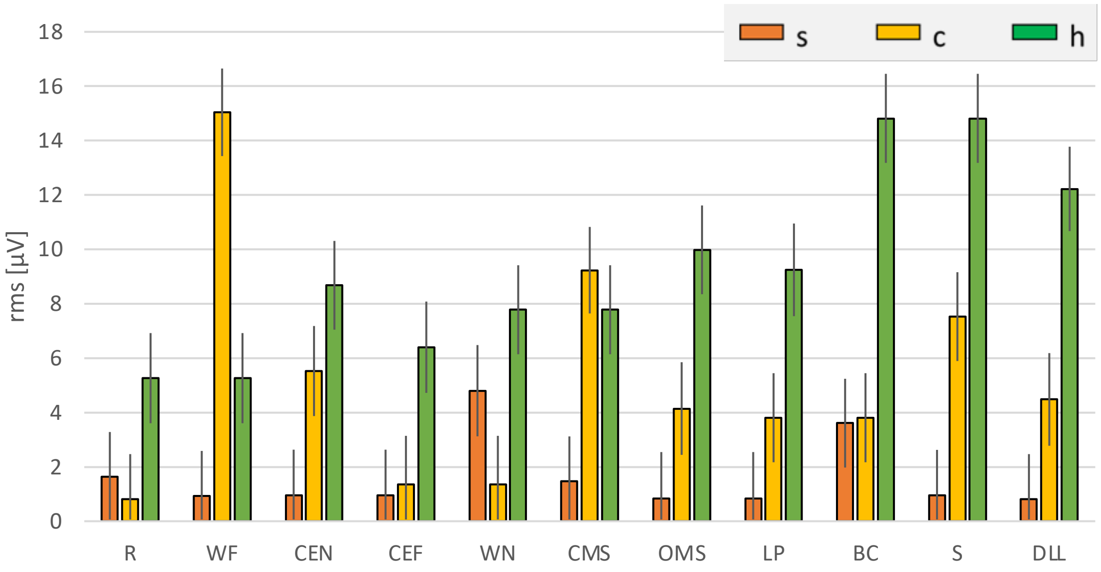


| | | | | | | | | | | | |
|---|---|---|---|---|---|---|---|---|---|---|---|
| s vs. c | | * | | *** | *** | * | | | *** | | *** |
| s vs. h | | ** | | *** | *** | | | *** | *** | | *** |
| c vs. h | | | | ** | | | | *** | | | |

# 15 - OOr

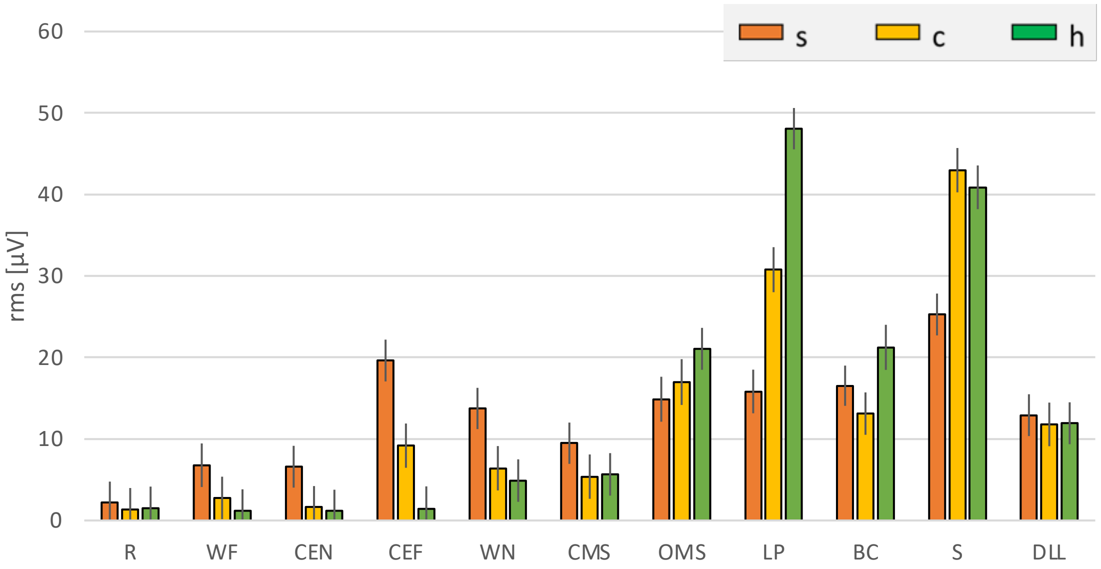


| | | | | | | | | | | | |
|---|---|---|---|---|---|---|---|---|---|---|---|
| s vs. c | | | * | *** | *** | | | *** | | *** | |
| s vs. h | | * | * | *** | *** | | ** | *** | | *** | |
| c vs. h | | | | *** | | | | *** | *** | | |

# 16 - Ment

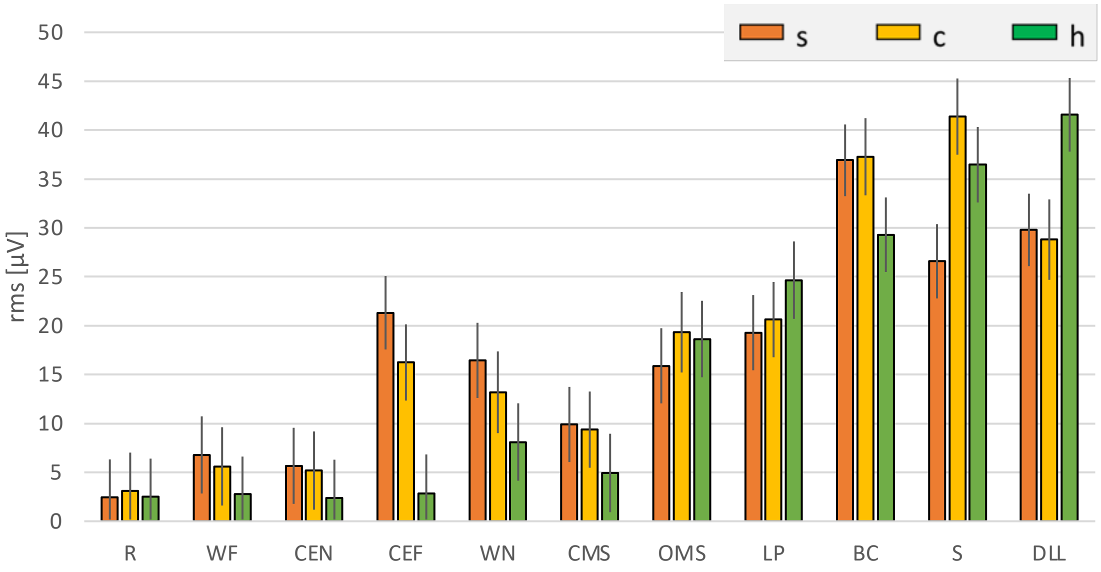


| | | | | | | | | | | | |
|---|---|---|---|---|---|---|---|---|---|---|---|
| s vs. c | | | | | | | | | | *** | |
| s vs. h | | | | *** | * | | | | * | ** | *** |
| c vs. h | | | | *** | | | | | * | | *** |

# 17 - Mass

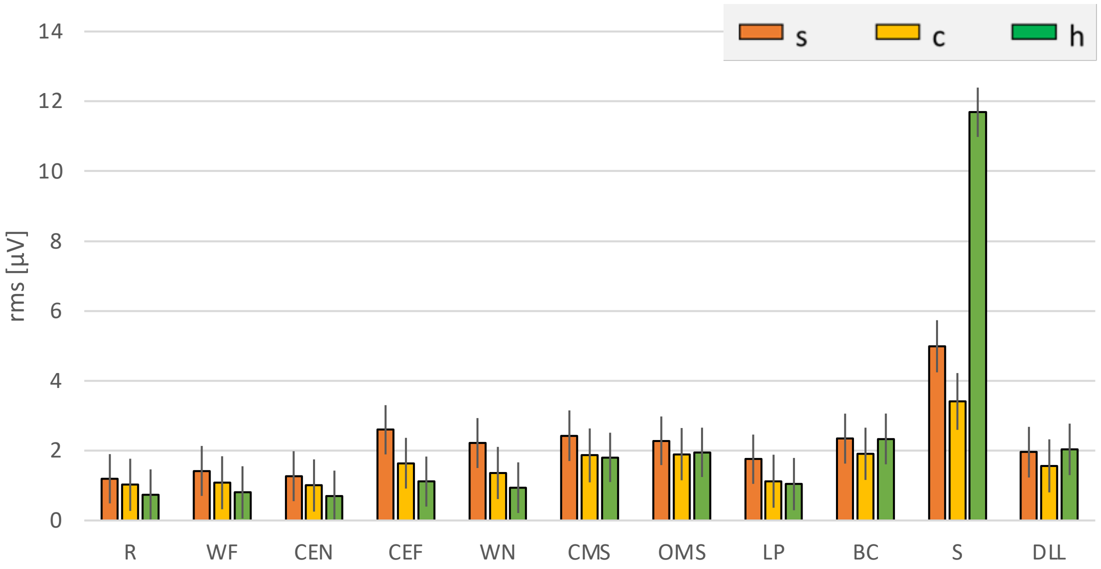


| | | | | | | | | | | | |
|---|---|---|---|---|---|---|---|---|---|---|---|
| s vs. c | | | | | | | | | | * | |
| s vs. h | | | | * | | | | | | *** | |
| c vs. h | | | | | | | | | | *** | |

# 18 - Zyg

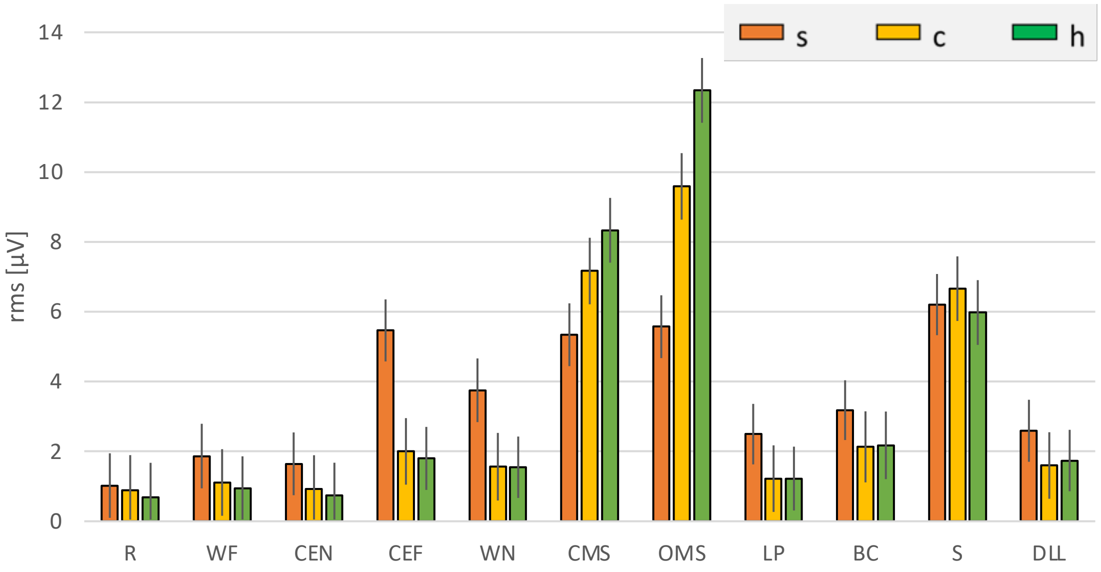


| | | | | | | | | | | | |
|---|---|---|---|---|---|---|---|---|---|---|---|
| s vs. c | | | | *** | ** | * | *** | | | | |
| s vs. h | | | | *** | ** | *** | *** | | | | |
| c vs. h | | | | | | | *** | | | | |

## 19 - LLS

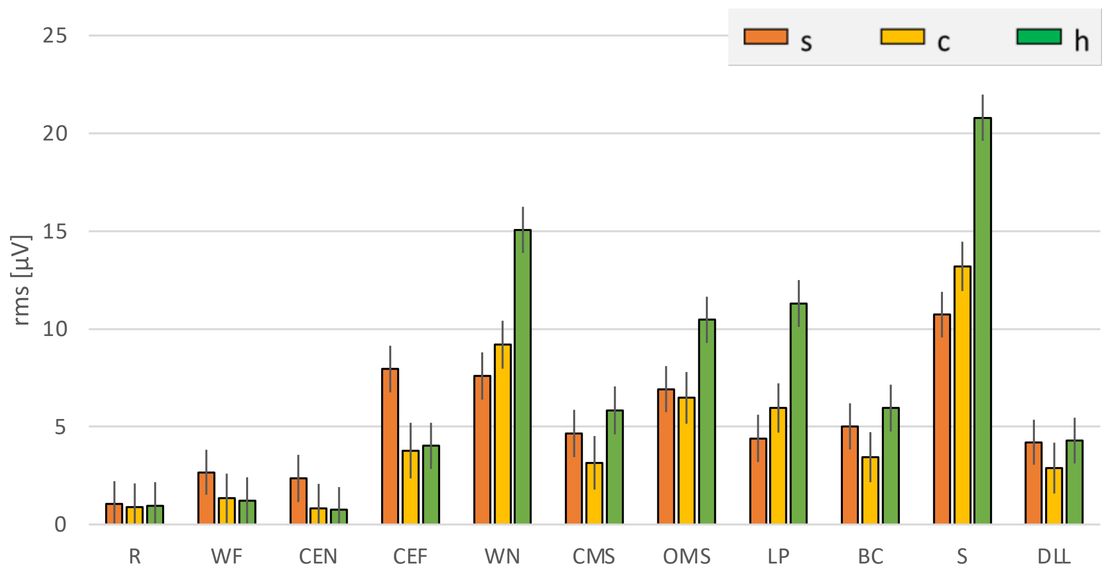


| | | | | | | | | | | | |
|---|---|---|---|---|---|---|---|---|---|---|---|
| s vs. c | | | | *** | | | | | | * | |
| s vs. h | | | | *** | *** | | *** | *** | | *** | |
| c vs. h | | | | | *** | * | *** | *** | * | *** | |

## 20 - OOc

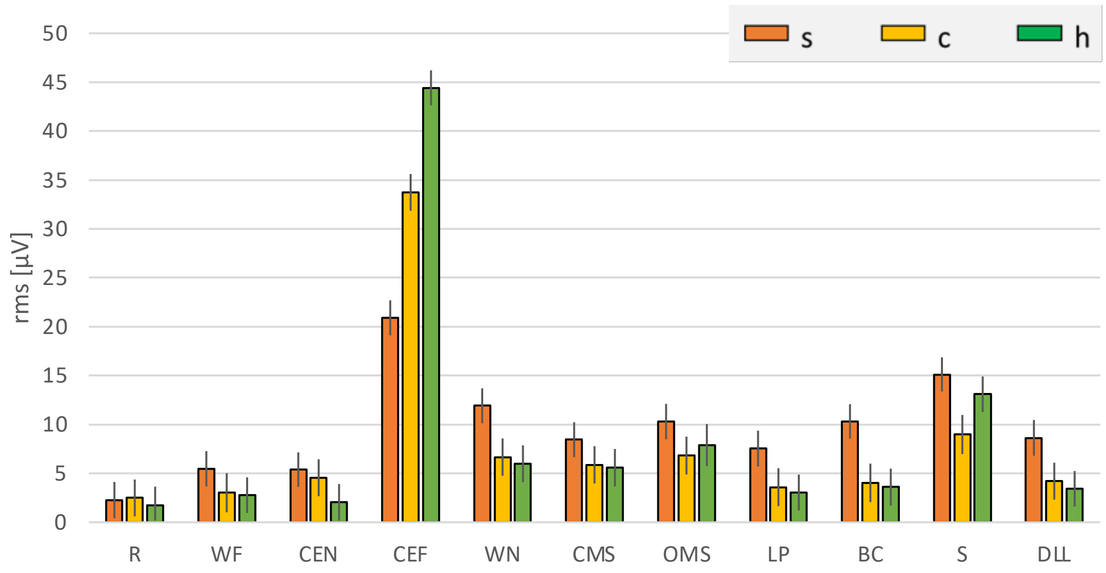


| | | | | | | | | | | | |
|---|---|---|---|---|---|---|---|---|---|---|---|
| s vs. c | | | | *** | *** | | * | * | *** | *** | ** |
| s vs. h | | | | *** | *** | | | ** | *** | | *** |
| c vs. h | | | | *** | | | | | | * | |

# 21 - LF

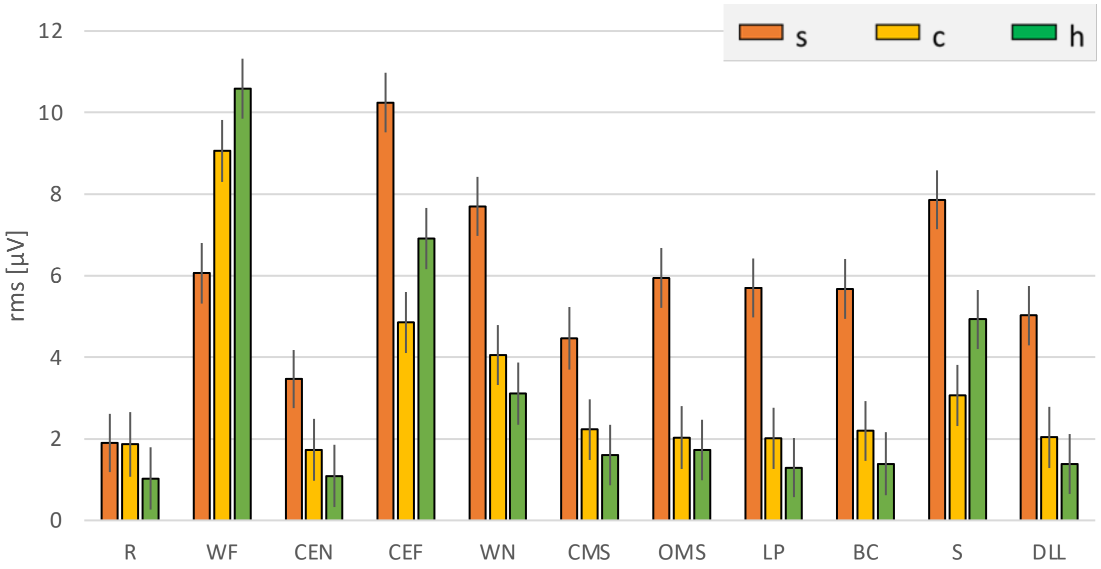


| | | | | | | | | | | | |
|---|---|---|---|---|---|---|---|---|---|---|---|
| s vs. c | | *** | *** | *** | *** | *** | *** | *** | *** | *** | *** |
| s vs. h | | *** | *** | *** | *** | *** | *** | *** | *** | *** | *** |
| c vs. h | | * | | ** | | | | | | ** | |

# 22 - MF

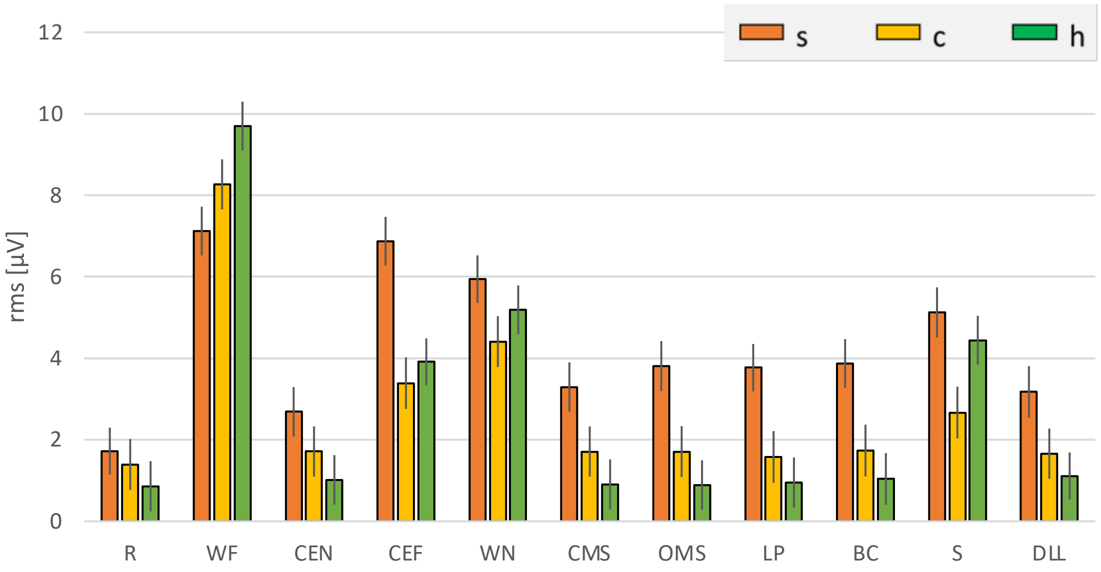


| | | | | | | | | | | | |
|---|---|---|---|---|---|---|---|---|---|---|---|
| s vs. c | | * | * | *** | *** | *** | *** | *** | *** | *** | ** |
| s vs. h | | *** | *** | *** | | *** | *** | *** | *** | | *** |
| c vs. h | | * | | | | | | | | *** | |

## 23 - Corr

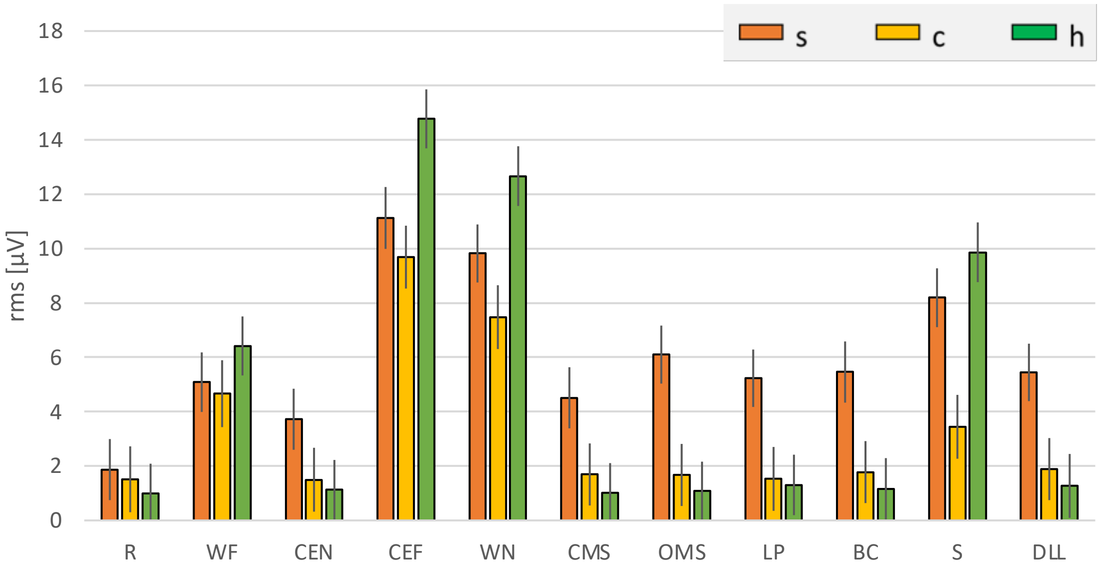


| | | | | | | | | | | | |
|---|---|---|---|---|---|---|---|---|---|---|---|
| s vs. c | | | * | | ** | ** | *** | *** | *** | *** | *** |
| s vs. h | | | ** | *** | ** | *** | *** | *** | *** | | *** |
| c vs. h | | | | *** | *** | | | | | *** | |

## 24 - DS

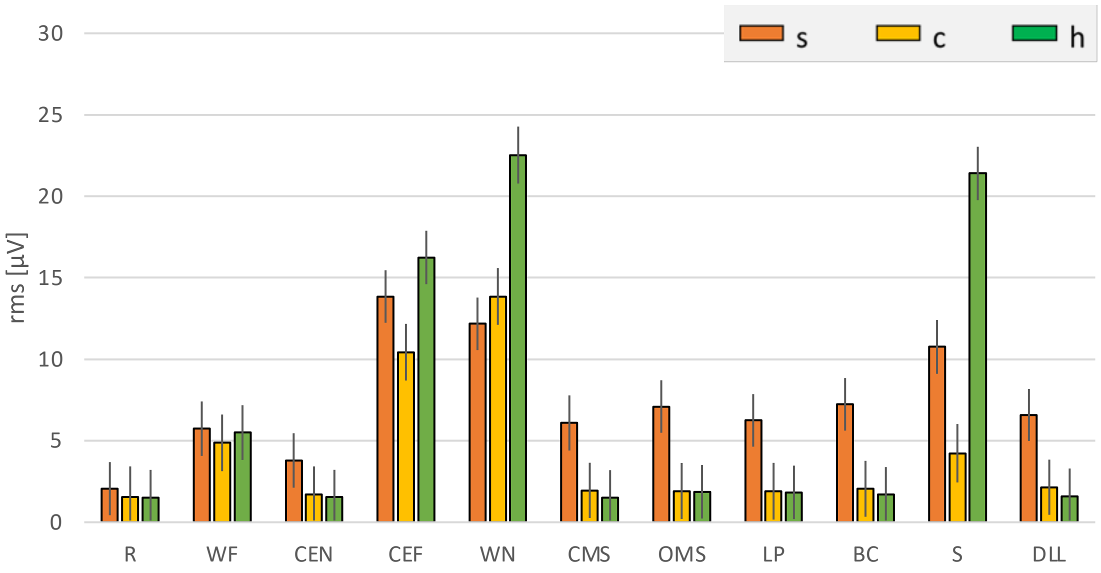


| | | | | | | | | | | | |
|---|---|---|---|---|---|---|---|---|---|---|---|
| s vs. c | | | | ** | | *** | *** | *** | *** | *** | *** |
| s vs. h | | | | | *** | *** | *** | ** | *** | *** | *** |
| c vs. h | | | | *** | *** | | | | | *** | |
