# Supplementary material for: Bilateral muscle activation in postparalytic facial synkinesis: a cross-sectional high-resolution surface electromyography study
Source: Sci Rep. 2026 Jan 14;16:2057. doi: 10.1038/s41598-026-36015-1 (PMC12808638; doi:10.1038/s41598-026-36015-1)
Supplement: Supplementary file 2 — Supplementary Material 2 [file 41598_2026_36015_MOESM2_ESM.pdf]

Supplementary Material for the Kuramoto Scheme

On the following pages, results are presented first as averages across all exercises, then for each exercise, and finally for each electrode position. Facial muscle activation is shown as root mean square (RMS) values in µV with mean ±95% confidence intervals. Statistical comparisons (corrected for multiple testing) are provided below each graph, with significant differences indicated by an asterisk (*$p<0.05$; **$p<0.01$; ***$p<0.001$, Holm–Bonferroni corrected) .

Kuramoto midline electrodes were not evaluated.

Abbreviations:

Electrode positions - Kuramoto scheme, excluding midline electrodes: E1/2, E3/4, E5/6, E7/8, E9/10, E13/14, E15/16, E17/18.

The facial movement tasks: R = Face at rest; WF = Wrinkling of the forehead; CEN = Closing the eyes normally; CEF = Closing the eyes forcefully; WN = Wrinkling of the nose; CMS = Closed mouth smiling; OMS = Open mouth smiling; LP = Lip puckering; BC = Blowing out the cheeks; S = Snarling; DLL = Depressing lower lip.

Facial sides: s = synkinetic side (orange); c = contralateral side (yellow); h = healthy controls (green)

Electrode schemes
Kuramoto electrodes E1-E24

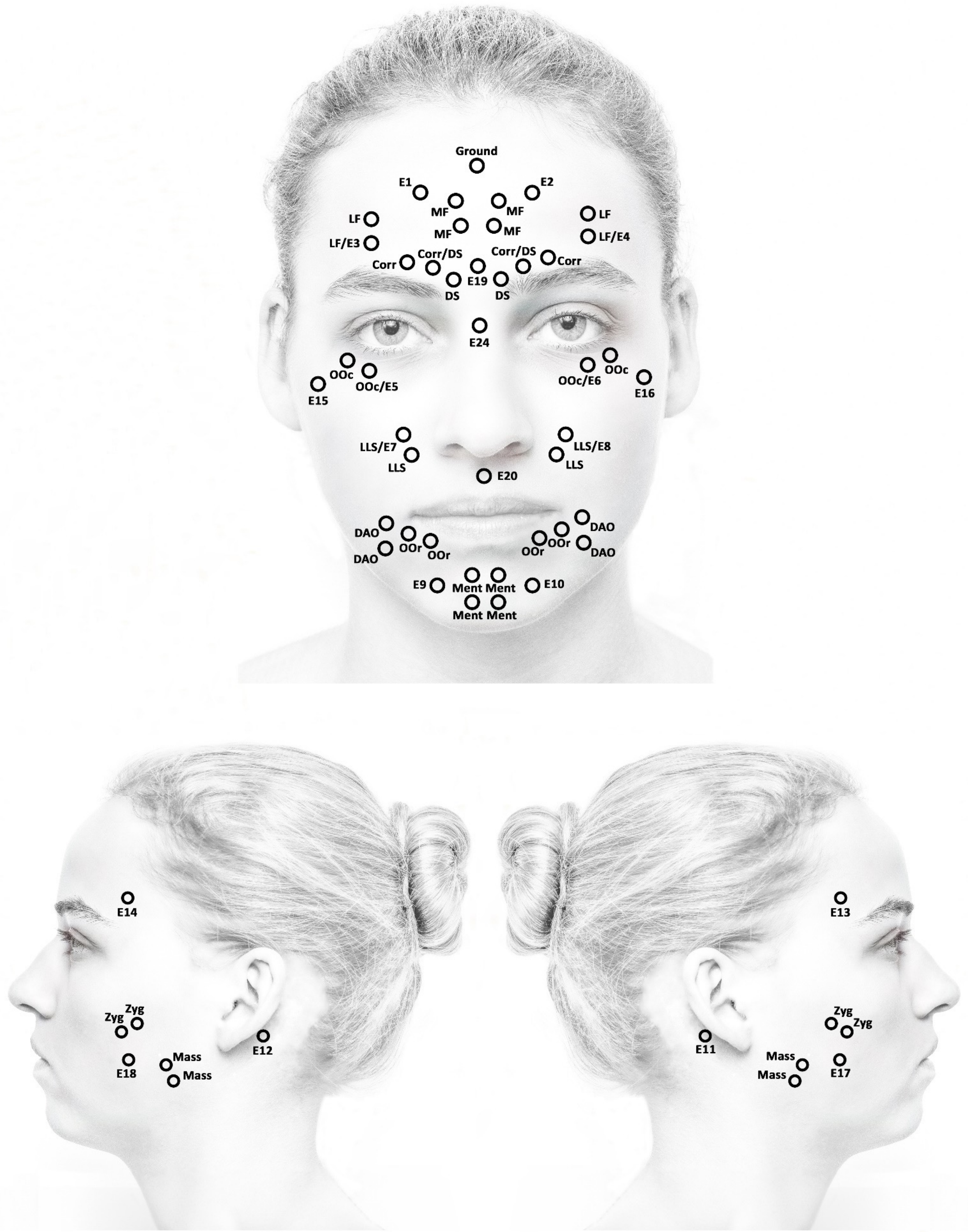

1 - Independent of facial expression

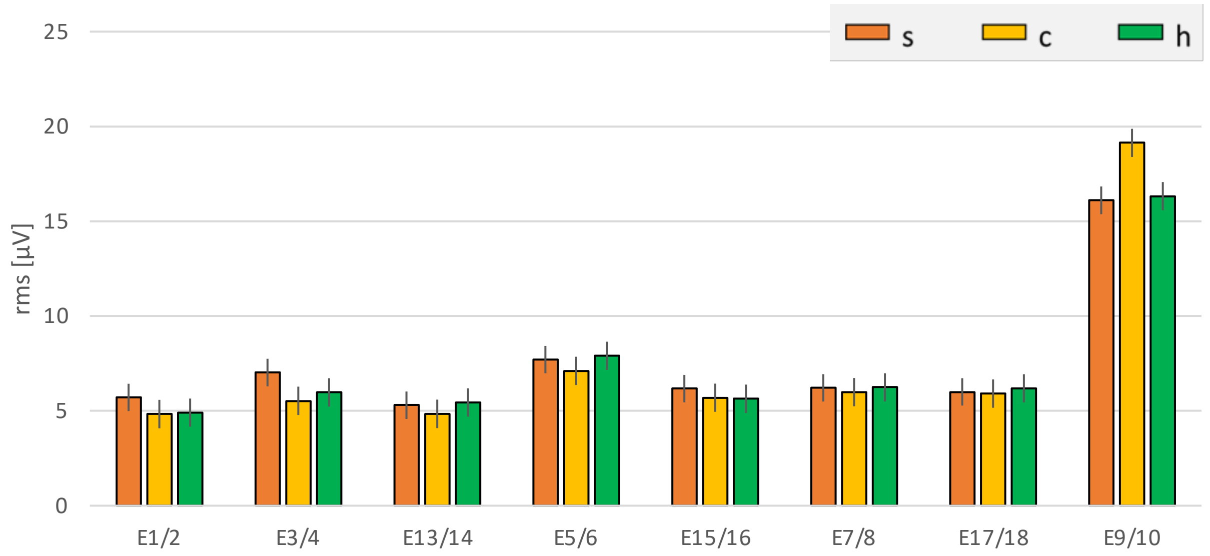

s vs. c		***						***
s vs. h								
c vs. h								***

2 - R

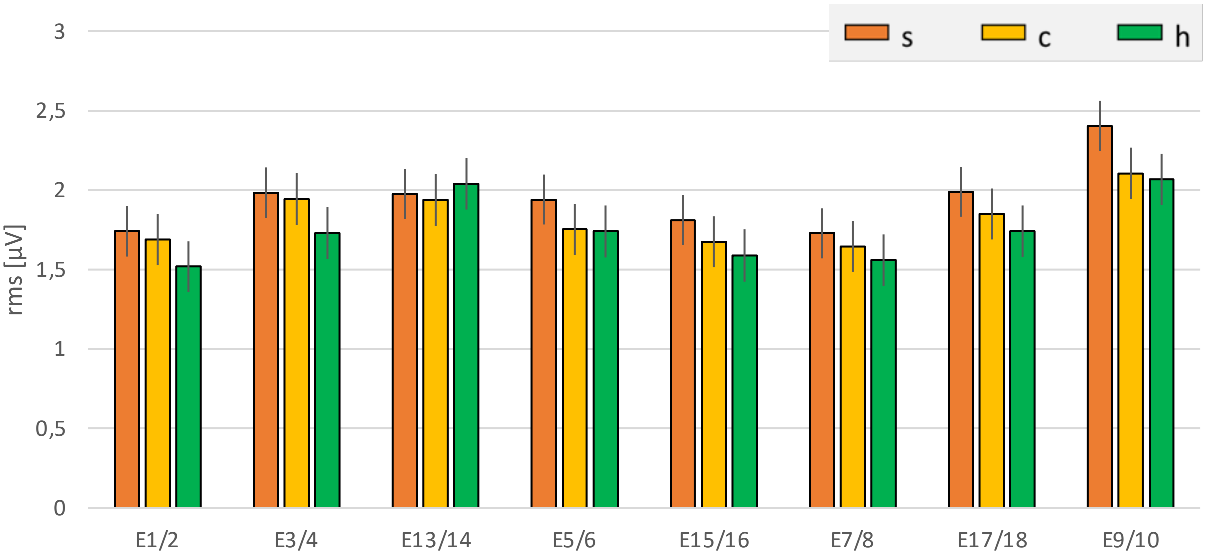

s vs. c								***
s vs. h								*
c vs. h								

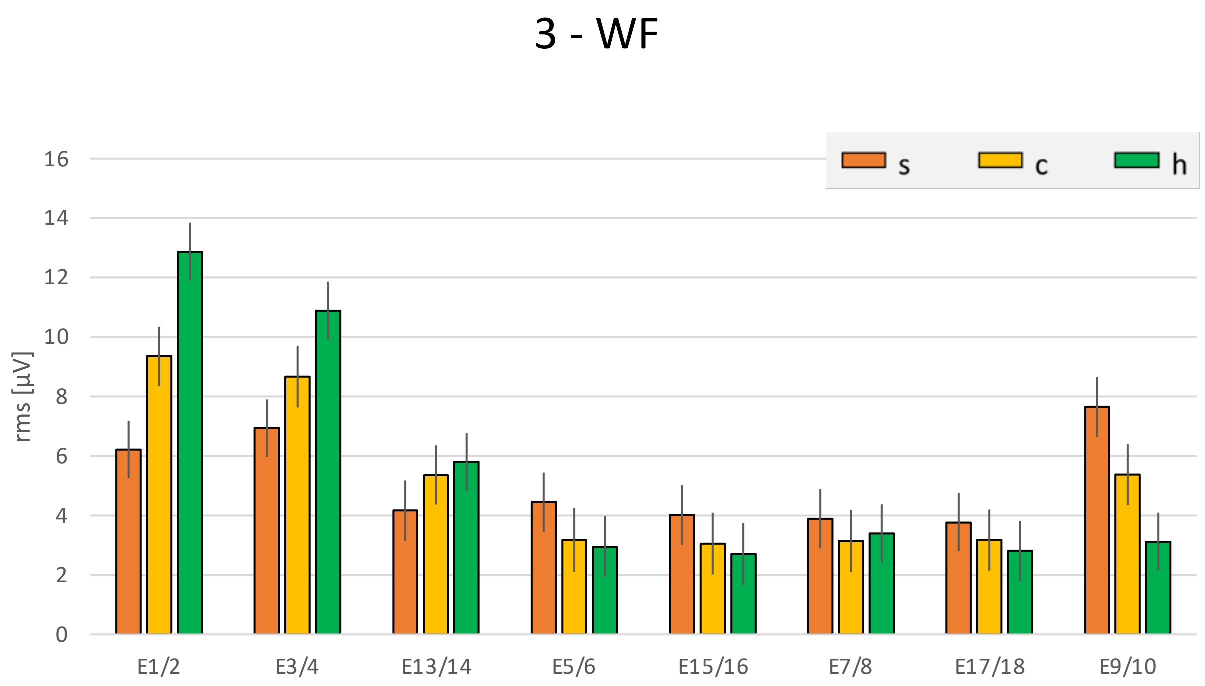

s vs. c	***	*						***
s vs. h	***	***						***
c vs. h	***	*						*

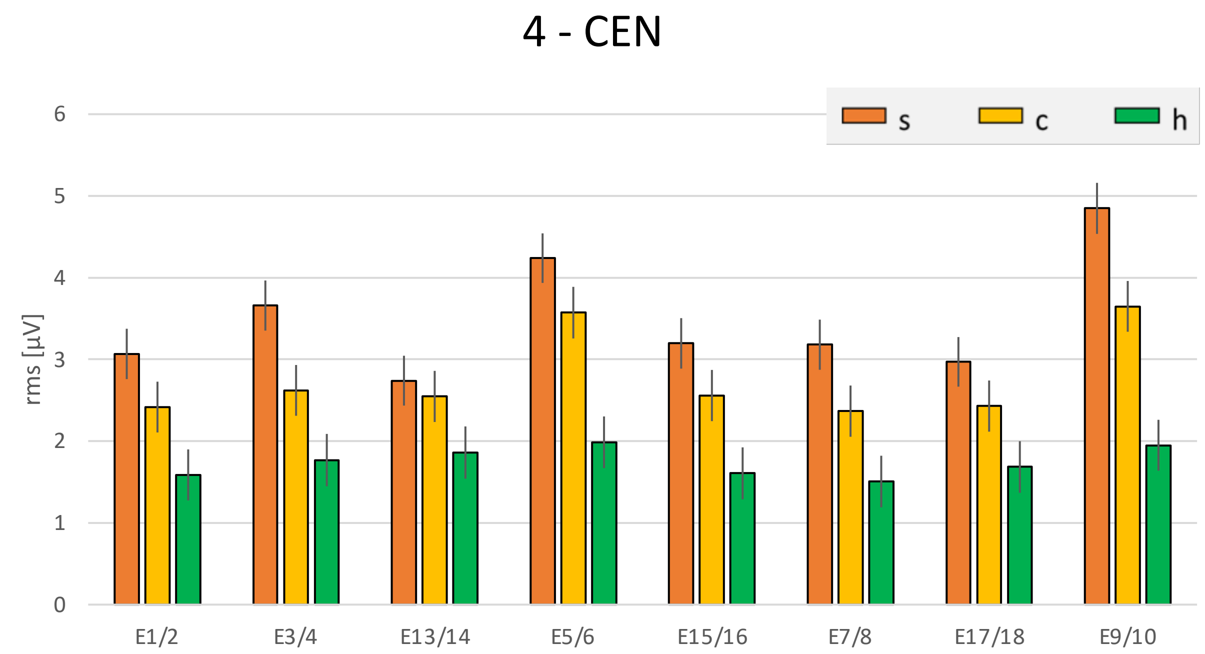

s vs. c	***	***		***	***	***	***	***
s vs. h	***	***	***	***	***	***	***	***
c vs. h	***	***	**	***	***	***	**	***

5 - CEF

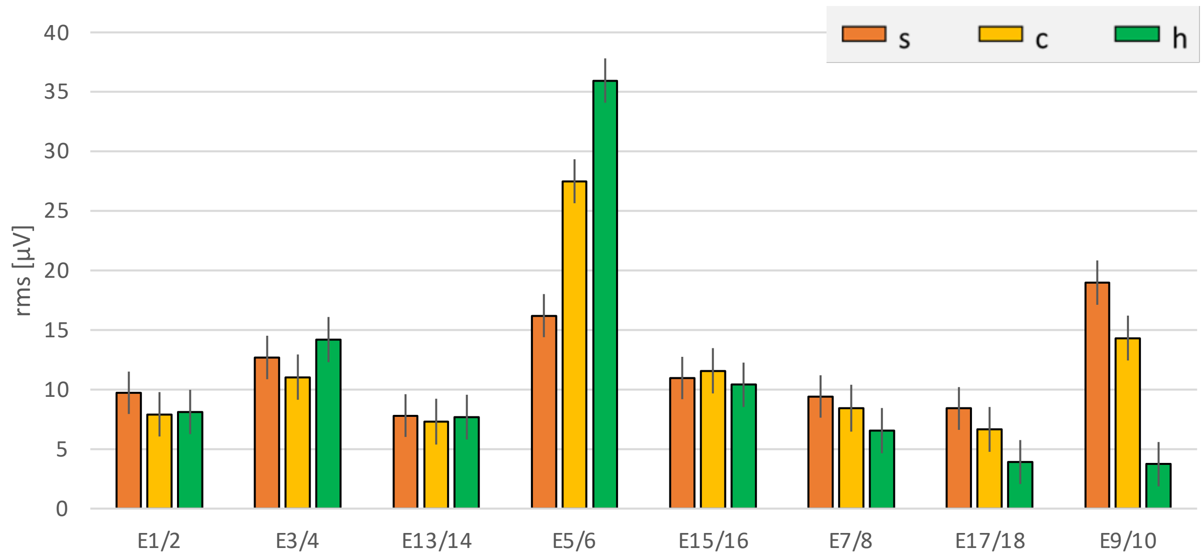

s vs. c				***				***
s vs. h				***			**	***
c vs. h				***				***

6 - WN

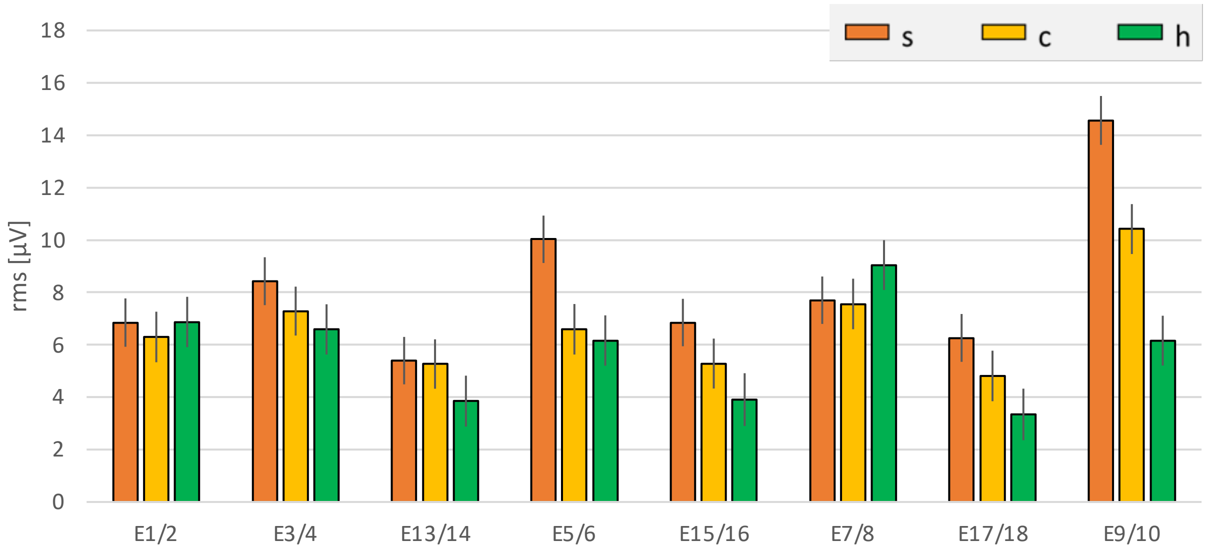

s vs. c				***	*		*	***
s vs. h		*		***	***		***	***
c vs. h								***

7 - CMS

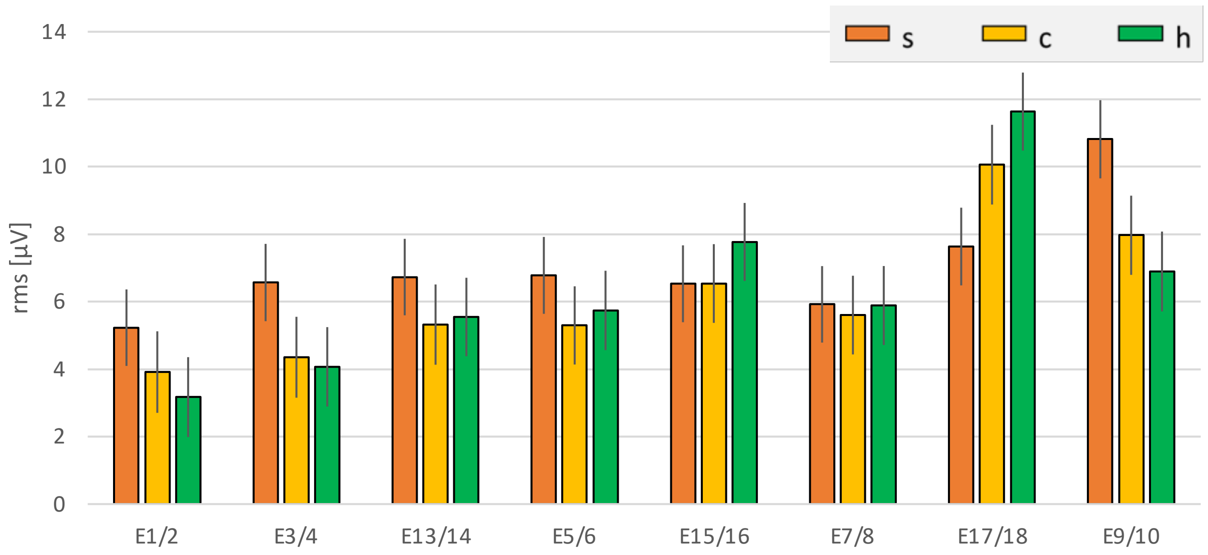

s vs. c	*	***	*	*			***	***
s vs. h		*					***	***
c vs. h								

8 - OMS

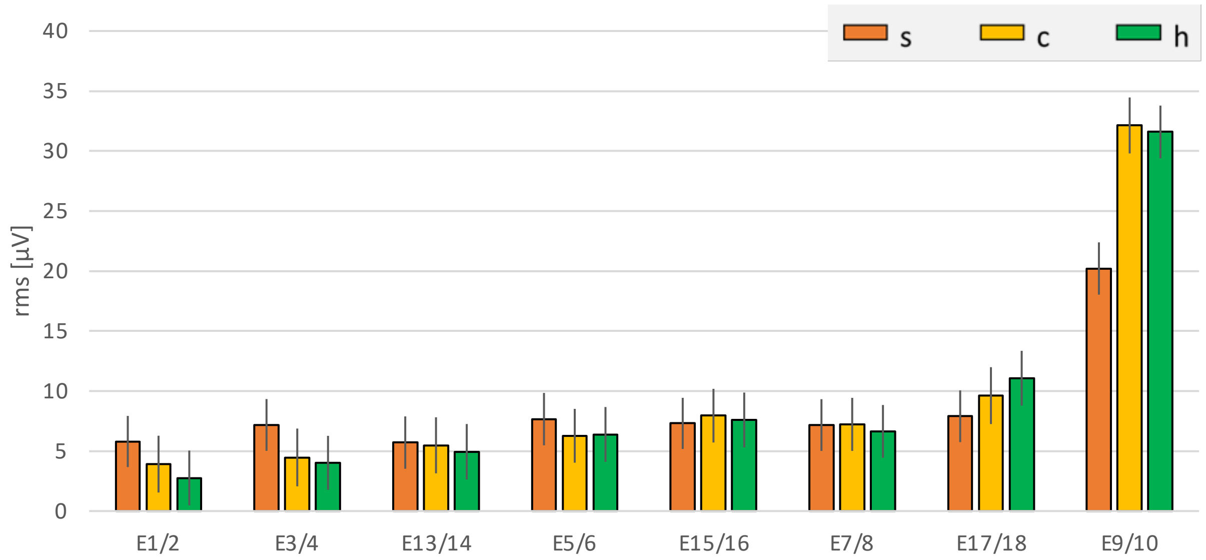

s vs. c								***
s vs. h								***
c vs. h								

9 - LP

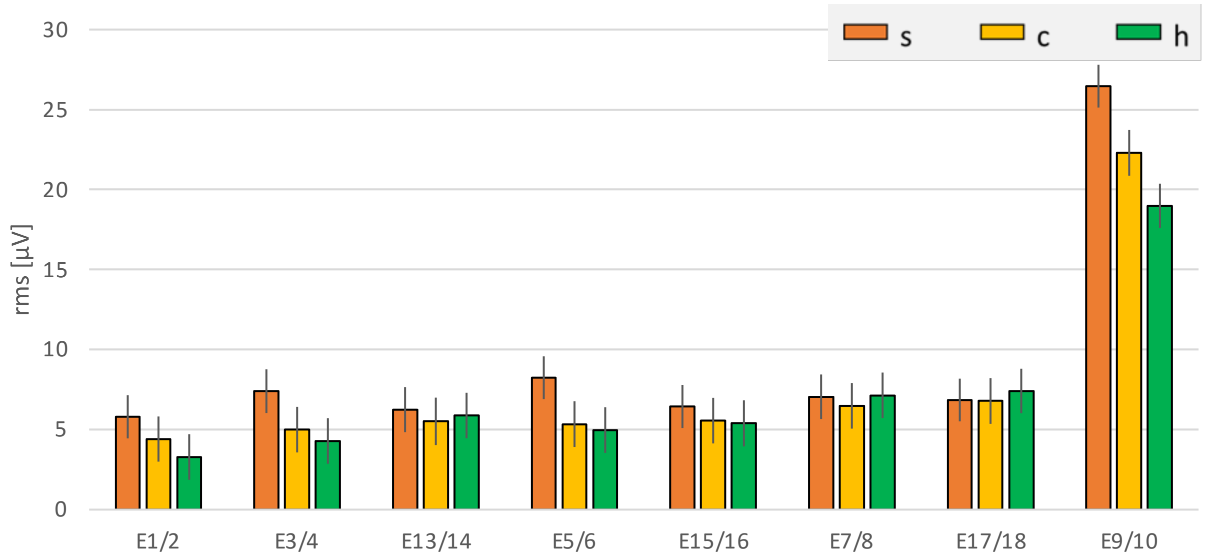

s vs. c		*		**				***
s vs. h		*		**				***
c vs. h								**

10 - BC

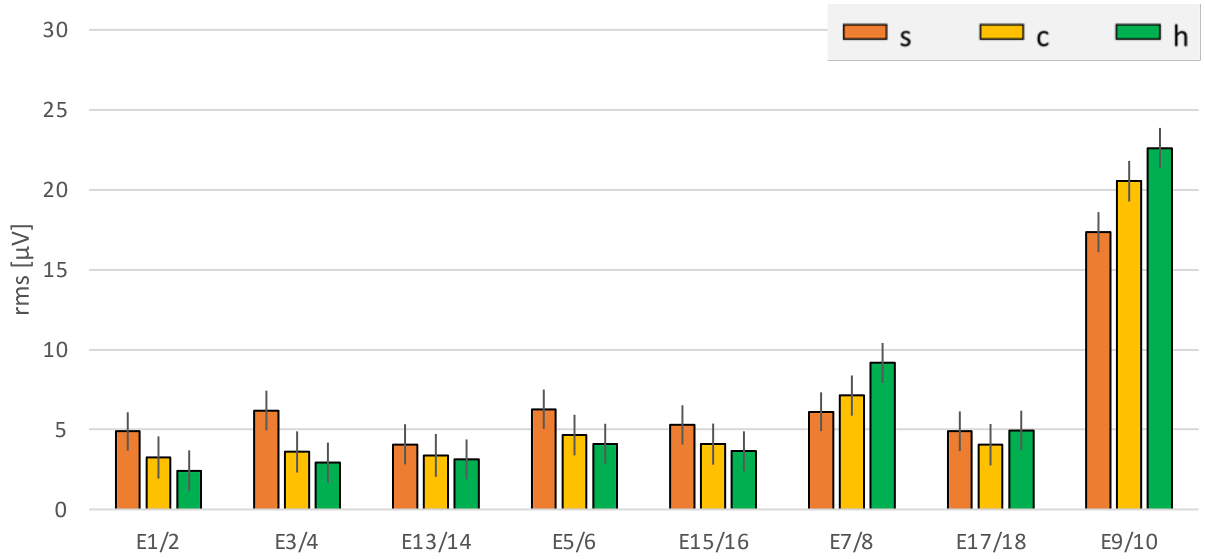

s vs. c		**						***
s vs. h	*	**				**		***
c vs. h								

11 - S

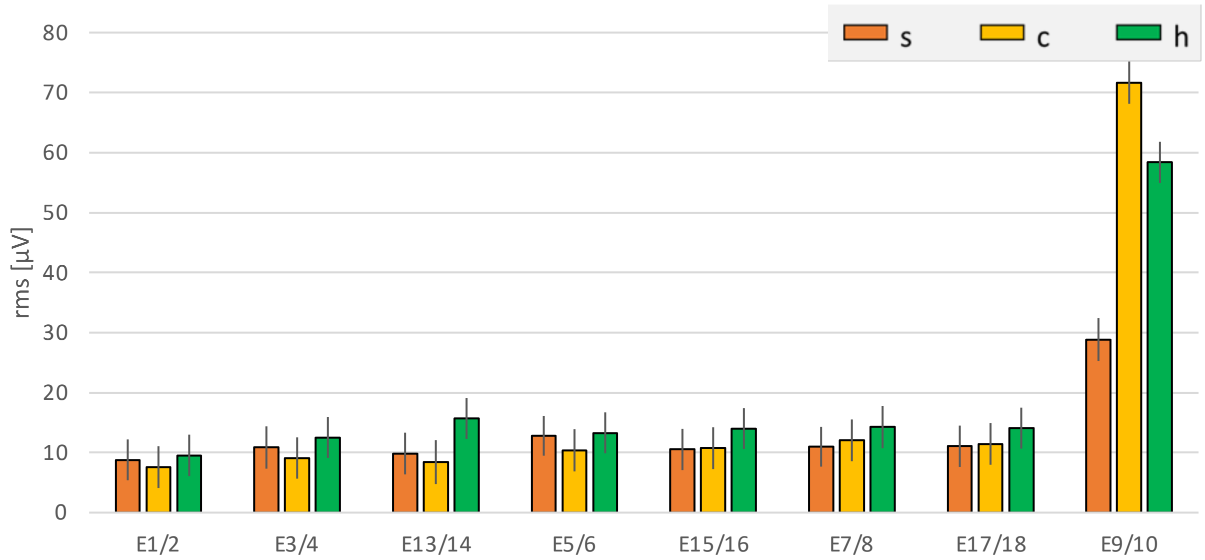

s vs. c								***
s vs. h								***
c vs. h			*					***

12 - DLL

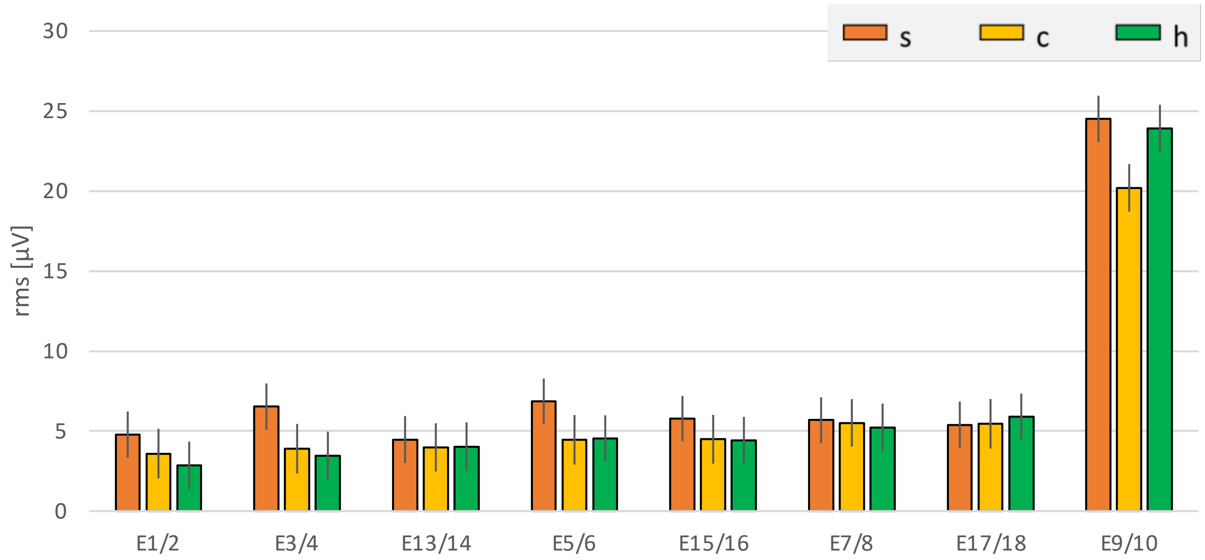

s vs. c								***
s vs. h		*						
c vs. h								**

13 - Independent of electrode position

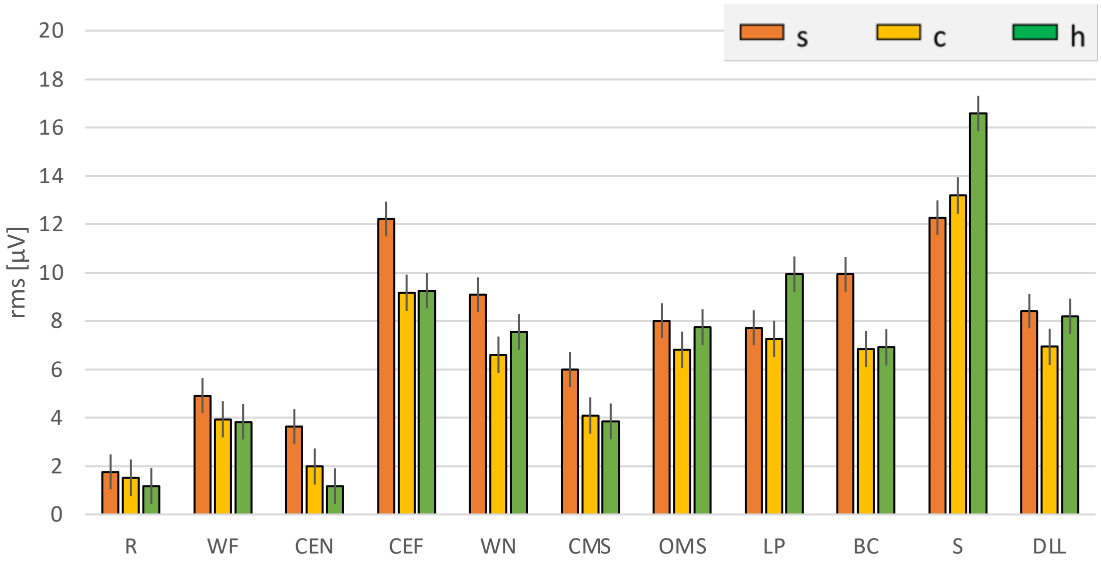

s vs. c					**				***	***	**
s vs. h			*		***				***	***	
c vs. h											

14 - E9/E10

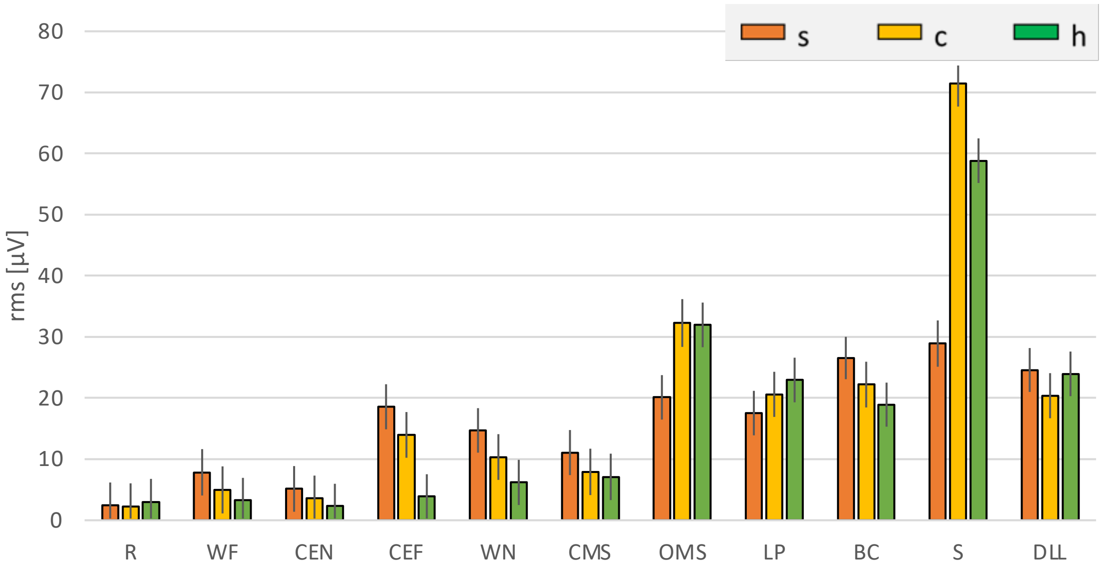

s vs. c							***			***	
s vs. h				***	*		***		*	***	
c vs. h				**						***	

15 - E17/E18

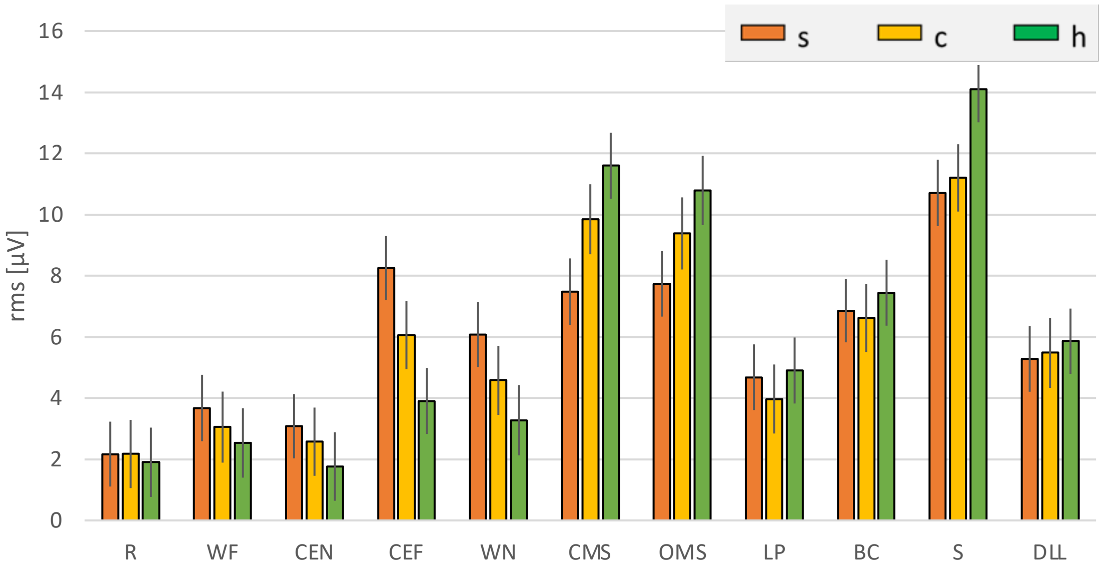

s vs. c				*		*					
s vs. h				***	**	***	**			***	
c vs. h										**	

16 - E15/E16

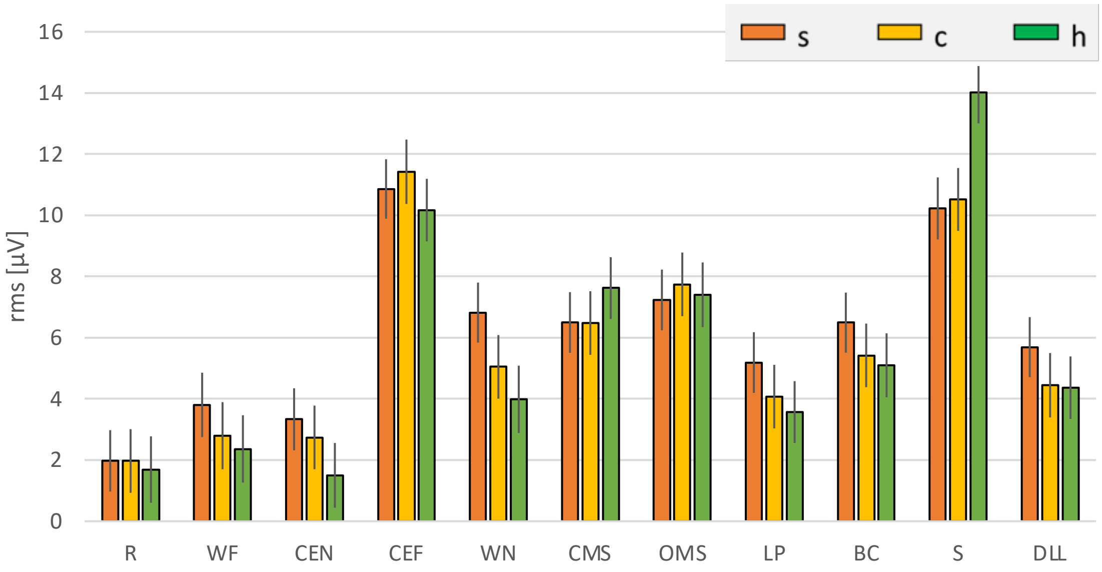

s vs. c											
s vs. h					**					***	
c vs. h										***	

17 - E7/E8

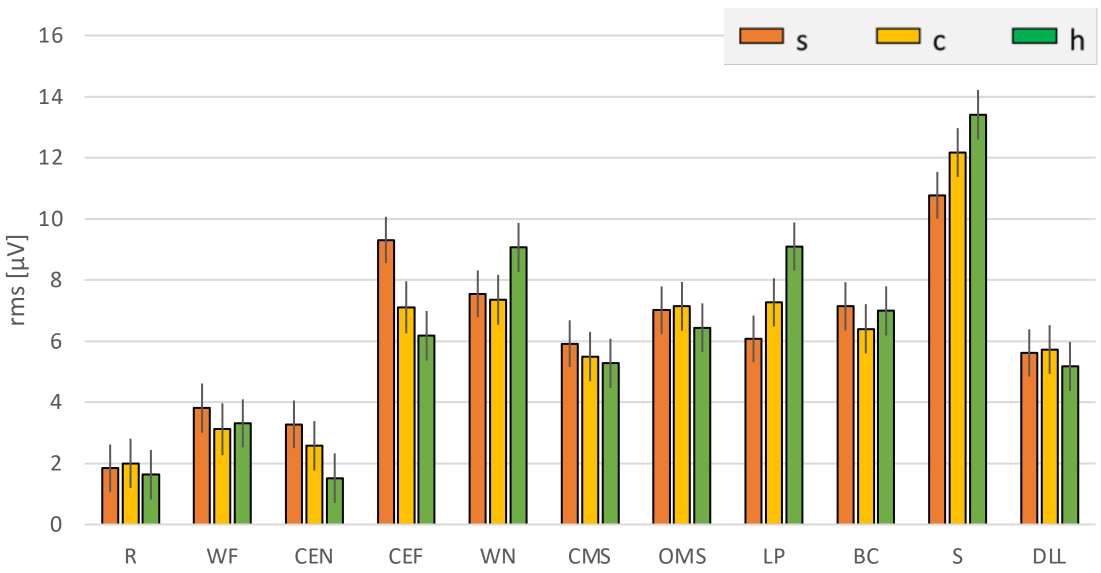

s vs. c				***						*	
s vs. h			*	***				***		***	
c vs. h					*			*			

18 - E5/E6

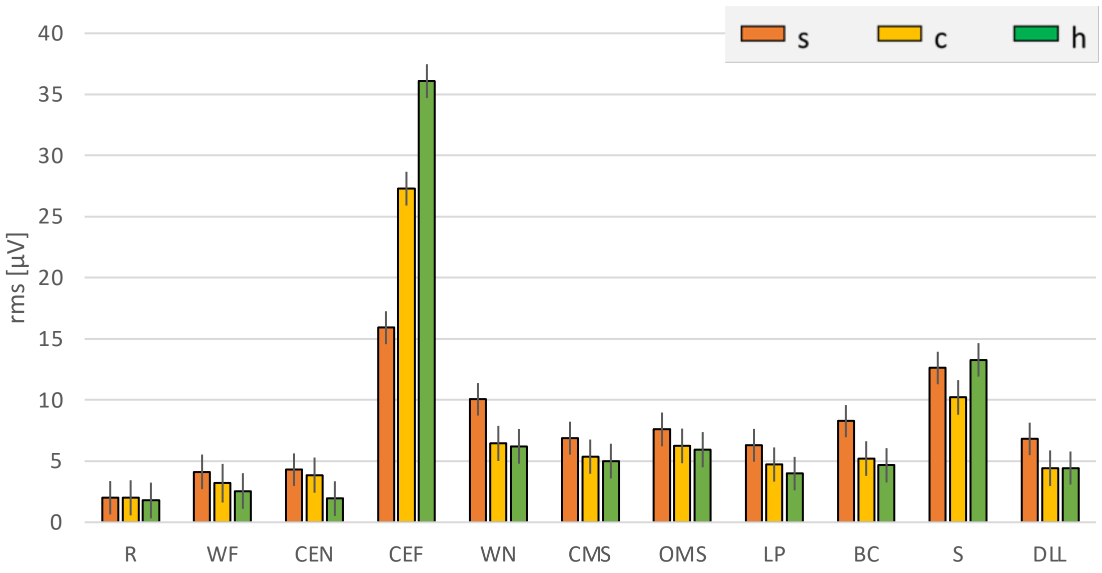

s vs. c				***	***				**		
s vs. h				***	**				**		
c vs. h				***						*	

19 - E13/E14

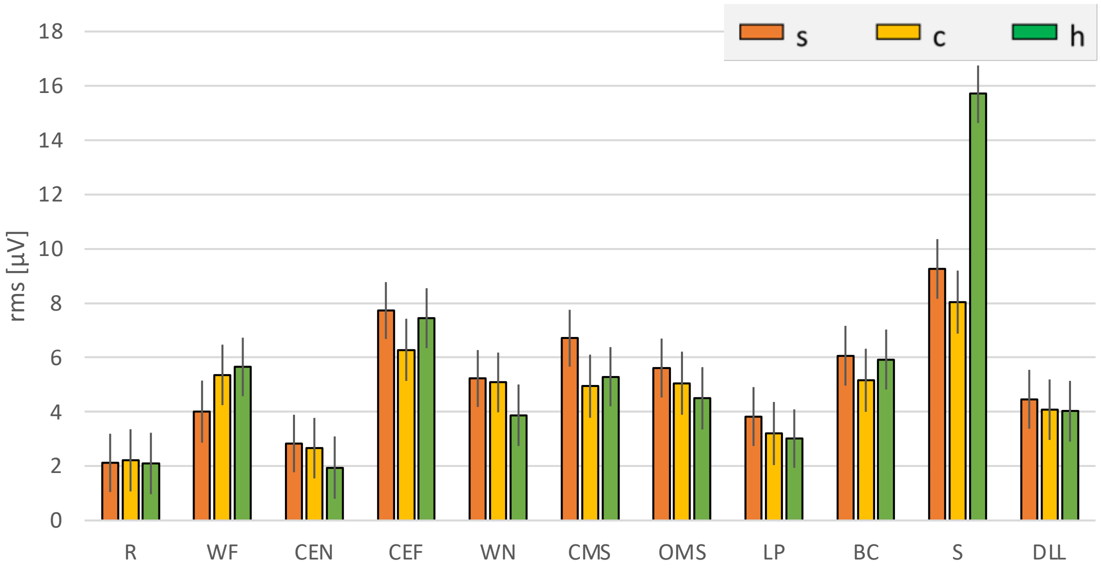

s vs. c											
s vs. h										***	
c vs. h										***	

20 - E3/E4

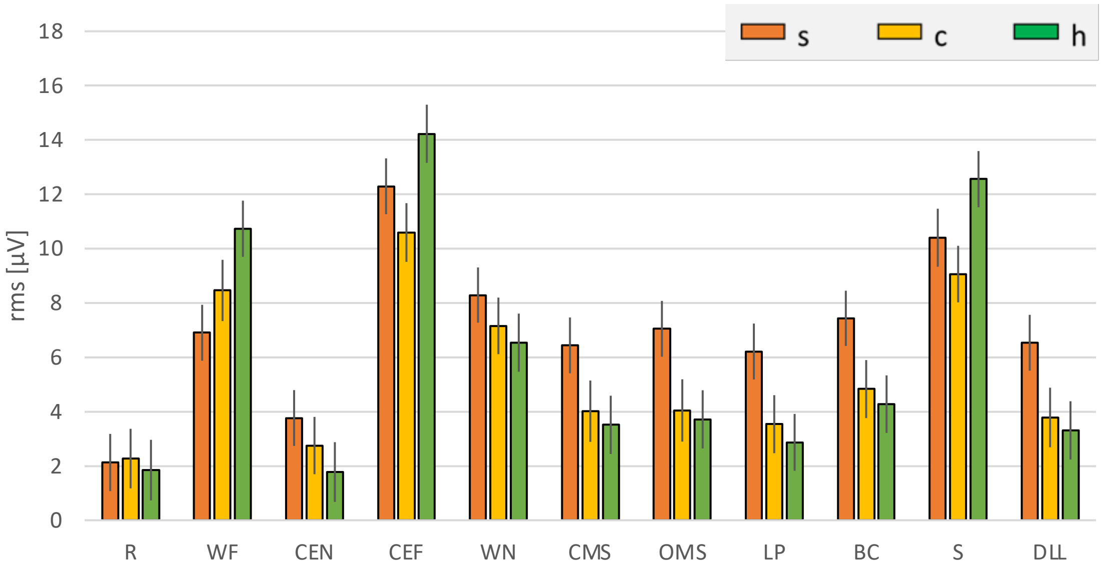

s vs. c						**	***	***	***		***
s vs. h		***	*	*	*	***	***	***	***	*	***
c vs. h		*		***						***	

21 - E1/E2

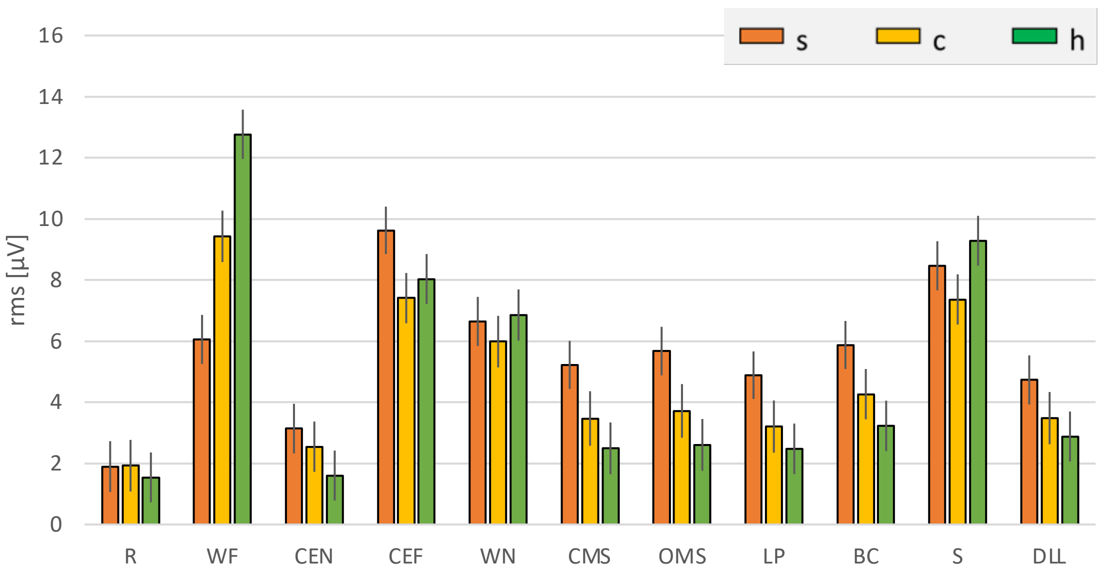

s vs. c		***		***		**	**	**	**		
s vs. h		***	*	*		***	***	***	***		**
c vs. h		***								*	
