# Supplementary material for: Bilateral muscle activation in postparalytic facial synkinesis: a cross-sectional high-resolution surface electromyography study
Source: Sci Rep. 2026 Jan 14;16:2057. doi: 10.1038/s41598-026-36015-1 (PMC12808638; doi:10.1038/s41598-026-36015-1)
Supplement: Supplementary file 3 — Supplementary Material 3 [file 41598_2026_36015_MOESM3_ESM.pdf]

The standardized facial exercises

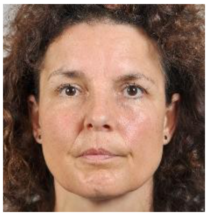

Face at rest

(R)

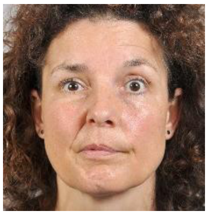

Wrinkling of the forehead

(WF)

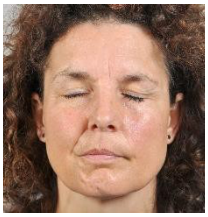

Closing the eyes normally

(CEN)

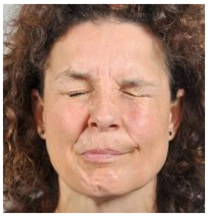

Closing the eyes forcefully

(CEF)

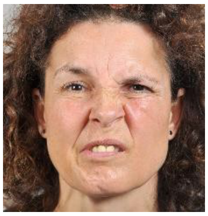

Wrinkling of the nose

(WN)

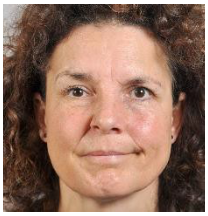

Closed mouth smiling

(CMS)

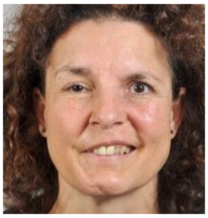

Open mouth smiling

(OMS)

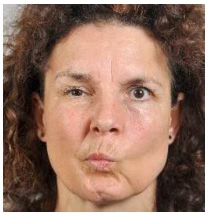

Lip puckering

(LP)

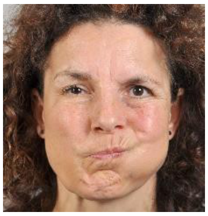

Blowing out the cheeks

(BC)

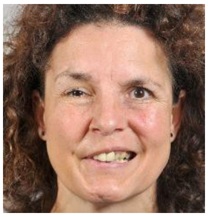

Snarling

(S)

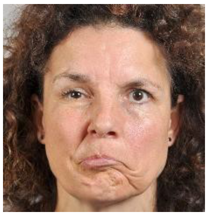

Depressing lower lips

(DLL)
